# Hypoxia-Driven Immune Escape in Clear Cell Renal Cell Carcinoma: A Prognostic Model and Dual-Functional Biomarker PLOD2 for Immunotherapy Stratification

**DOI:** 10.7150/jca.114151

**Published:** 2026-01-01

**Authors:** Fei Xiao, Yi Guan, Huajie Song, Wan Xiang

**Affiliations:** 1 Department of Urology, Renmin Hospital of Wuhan University, Wuhan, China.; 2 Department of Biological Repositories, Zhongnan Hospital of Wuhan University, Wuhan, China.

**Keywords:** ccRCC, tumor Microenvironment, immunotherapy response, hypoxia, risk model, PLOD2

## Abstract

For patients with recurrent or metastatic clear cell renal cell carcinoma (ccRCC), immunotherapy has demonstrated substantial antitumor activity. However, accurately predicting which patients will benefit from these therapies remains a major challenge. This study aims to elucidate the regulatory role of the hypoxic tumor microenvironment in immune suppression and immune escape, to develop a hypoxia-based prognostic model, and to identify key biomarkers to guide personalized treatment decisions. We applied weighted gene co-expression network analysis (WGCNA) to screen hypoxia-related genes and constructed a hypoxia risk score (HRS) model using LASSO-Cox regression. We found that the HRS model effectively predicted immunotherapy response and prognosis, with patients in the high-HRS group exhibiting significantly shorter overall survival. A high HRS was associated with immune escape by reshaping the T-cell-infiltrated tumor microenvironment (TME), and showed strong positive correlations with cancer-immunity cycle activity, PD-L1/CTLA-4 immune checkpoint expression, and T-cell inflammation scores. Importantly, cell-based and animal experiments demonstrated that PLOD2, a key gene in the HRS model, plays a critical role in hypoxia-induced immune escape in ccRCC. PLOD2 significantly promoted ccRCC cell growth and migration *in vitro* and *in vivo*. High PLOD2 expression in clinical samples was associated with ccRCC progression and potentially enhanced sensitivity to immunotherapy by modulating tumor mutational burden and immune escape-related pathways. In summary, our study successfully constructed an HRS model to predict the efficacy of immune checkpoint inhibitor (ICI)-based immunotherapy. PLOD2 was identified as a dual-functional biomarker with both prognostic and predictive value for immunotherapy. The HRS model provides a quantitative tool for immunotherapy stratification. Notably, high PLOD2 expression indicates tumor progression yet paradoxically associates with enhanced immunotherapy response through activation of immune escape pathways, thereby offering a potential therapeutic target for converting "cold tumors" into “hot tumors”.

## Introduction

Clear cell renal cell carcinoma (ccRCC) is the most common subtype of kidney cancer, and its incidence is increasing 1. For patients with recurrent or metastatic ccRCC, immunotherapy has shown unprecedented antitumor activity 2. A subset of patients with ccRCC may benefit from immunotherapy, which improves their quality of life and may extend overall survival. However, in most cases, immunotherapy does not provide durable clinical benefit to patients with ccRCC 3. Therefore, developing effective models to predict immune checkpoint blockade (ICB) response in ccRCC, based on comprehensive characterization of the tumor microenvironment (TME), is crucial for improving treatment outcomes and guiding the development of targeted therapies.

According to large-scale tumor transcriptomic analyses, ccRCC is characterized by high levels of immune infiltrate; however, poor outcomes after nephrectomy are associated with high immune infiltration 4. High abundance of B-cells and T-cells have been reported as features of patients who respond to atezolizumab (anti-PD-L1) and nivolumab (anti-PD-1) 5, 6. Nevertheless, cross-validation of these features as biomarkers has failed to yield consistent results, possibly due to tumor heterogeneity 7. Immune cells, vascular cells and fibroblasts together constitute the ccRCC tissue. The tumor vasculature and fibroblasts undergo structural and functional alterations, which adversely affect O_2_ diffusion and perfusion, leading to local hypoxia 8. Several studies reported that hypoxia was a hallmark of tumorigenesis, invasion, and metastasis 9, 10. In the natural antitumor immune response, adequate oxygen levels are essential, as hypoxia inhibits the activity of natural killer (NK) cells and cytotoxic T lymphocytes (CTLs) 11. Additionally, hypoxia modulates tumor-associated macrophages and regulatory T lymphocytes and increases the expression of immunosuppressive factors (TGFB, TIGIT, CD96, and IL10), thereby promoting immunosuppression and tumor immune escape 12, 13. In recent years, immune checkpoint inhibitors targeting PD-1/PD-L1 and CTLA-4 have been increasingly used in clinical practice 14, 15. Therefore, the hypoxic status, immune landscape, and overall microenvironment of ccRCCs warrant further investigation.

Recent advances in immuno-oncology have highlighted the complexity of the tumor immune microenvironment (TIME) and its regulation by metabolic, inflammatory, and cellular factors. The Warburg effect and aerobic glycolysis have been identified as key metabolic drivers of tumor progression and immune evasion, often regulated by non-coding RNAs and oncogenic pathways such as PI3K/AKT and HIF signaling 16. Hypoxia-inducible factors (HIFs), particularly HIF-1, mediate adaptive responses to oxygen deprivation and promote angiogenesis, Epithelial-Mesenchymal Transition (EMT), and immunosuppressive signaling in solid tumors 17. Under hypoxic conditions, HIF-1α stabilization via inhibition of proline hydroxylation leads to transcriptional activation of glycolytic enzymes (e.g., LDHA, PKM2), resulting in lactate accumulation that suppresses effector T-cell function while promoting regulatory T-cell (Treg) and myeloid-derived suppressor cell (MDSC) expansion 17. This metabolic reprogramming not only fuels tumor proliferation but also depletes oxygen and glucose in the TME, thereby fostering immune evasion through upregulated immunosuppressive metabolites like adenosine 18. Advances in multi-omics platforms, such as TIMER, have enabled the exploration of immune infiltration patterns across tumor types 19. Moreover, cytokines such as TNF-α, with context-dependent effects on tumor survival or suppression, underscore the dynamic crosstalk within the TME 20. The TME also orchestrates processes such as EMT and therapy resistance, offering novel targets for intervention 21. In this landscape, cytotoxic lymphocytes—including CD8⁺ CTLs, NK, and γδ T cells—play pivotal roles in anti-tumor immunity, although challenges in defining biomarkers and reversing their exhaustion remain 22, 23. These insights collectively emphasize the urgent need for robust biomarkers that reflect the functional and metabolic status of the immune microenvironment to optimize immunotherapeutic strategies. In the present study, we have established a scoring system, called hypoxia risk score (HRS). The HRS was effective in predicting the overall survival (OS) of ccRCC patients in several independent cohorts. Integrated analyses indicated that HRS was strongly correlated with clinicopathological characteristics, molecular subtypes, somatic mutational landscape and immune cell infiltration. We found that HRS efficiently predicted the clinical benefit of immune checkpoint inhibitor (ICI)-based immunotherapy. In summary, we developed a novel HRS that has a potential prognostic value for ccRCC patients and may facilitate personalized counselling for immunotherapy.

## Materials and Methods

### Preprocessing and retrieval of data

The Cancer Genome Atlas (TCGA) data was acquired from the UCSC Xena data portal 24, which included pan-cancer RNA sequencing (RNA-seq) data somatic mutation data, and survival data. The RNA-seq data is converted using log2, and the somatic mutation data is examined using VarScan2 before being utilized to generate TMB (tumor mutational burden). The GISTIC technique is used to process copy number variation (CNV) data, which may be obtained through the UCSC Xena database. Table S1 contains abbreviations for many forms of cancer. Four ccRCC cohorts, GSE40355, GSE53757, GSE73731, and E-MTAB-1980, were downloaded from Gene Expression Omnibus (GEO) and EMBL's European Bioinformatics Institute (EMBL-EBI). The immunotherapy cohort for renal cell cancer was also gathered from the study's supplemental information 25. The tissue microarrays (TMA) for ccRCC were purchased from OUTDO Biotech (Shanghai, China), which included 150 ccRCC specimens and 30 surrounding normal tissues. The Joint Commission on Cancer Staging Manual (7th edition) was used to calculate TNM staging, and pathological grading was assessed using the Fuhrman grading system. Based on corresponding gene sets retrieved from Molecular Signature Database (MSigDB), a single-sample gene set enrichment analysis (ssGSEA) was used to quantify the levels of cancer-related hallmarks such as "Epithelial-mesenchymal transition (EMT)" and "Hypoxia" in each sample 26. The gene set of stemness was obtained from a previously published literature report 27.

### Weighted gene co-expression network and its modules

WGCNA is a systems biology technique based on high-throughput expression of genes 28. The β parameter is a soft threshold power parameter that enhances strong correlations and penalizes weak correlations between genes. A hierarchical clustering tree is constructed on this basis, with each branch of the tree representing a different gene module. The adjacency matrix is converted into a topological overlap matrix. On this basis, the genes were classified using the TOM method. The correlation between the model and the hypoxia score was assessed using Pearson correlation factors. The module with the greatest average gene significance was chosen as a possible model related to hypoxia.

### The development and validation of a HRS model

A random division of the TCGA KIRC (Kidney Renal Clear Cell Carcinoma) samples into training and validation sets was being conducted to determine the best genes for risk (7:3). A hypoxia risk score (HRS) model was built to predict prognosis of ccRCC patients in the training cohort based on prognostic indicators for 14 ccRCC-specific hypoxia genes. An algorithm was developed to calculate a risk score based on gene expression: 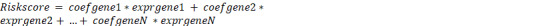, where N is the number of prognostic gene signatures, expr signifies the gene signature's expression profile, and coefgene denotes the gene signature's estimated regression coefficient from the LASSO Cox regression analysis. Based on the median value, the gene signature enables calculation of a risk score for each patient as well as the classification of patients into two risk groups (high risk and low risk). The "glmnet" package was used to perform the LASSO Cox regression analysis. A time-dependent receiver operating characteristic (ROC) analysis was conducted to determine the accuracy of the gene signature prediction. We used the area under the curve (AUC) as a predictor of accuracy, and we utilized the R package "timeROC" for time-dependent ROC analysis.

### Methods for assessing infiltrating immune cells

Single Sample Gene Set Enrichment Analysis (ssGSEA) is a non-parametric, unsupervised algorithm used to analyze changes in pathway and biological process activities in individual samples from a gene expression dataset. For this study, ssGSEA was applied to evaluate immune cell infiltration in the tumor microenvironment (TME) of ccRCC. The gene sets for TME-infiltrating immune cells were derived from the studies of Bindea *et al.* 29 and Charoentong *et al.* 30, and then merged (Table S10). The immune cell types included in the analysis were: innate immune cells including dendritic cells (DCs), eosinophils, mast cells, macrophages, natural killer cells, neutrophils. Adaptive immune cells including B cells, T cells, T helper cells, CD8+ T cells, regulatory T cells (Treg), and cytotoxic T cells. The Normalized Enrichment Score (NES) computed by ssGSEA was used to indicate the relative abundance of each TME-infiltrating immune cell type in BLCA. Additionally, the infiltration of endothelial cells and fibroblasts was assessed using the MCPcounter package in R software. To ensure robustness in immune infiltration estimation, we applied seven widely used deconvolution methods. Given the inherent differences among algorithms, only immune cell types showing consistent directional changes across at least four methods were considered high-confidence signals. This approach aligns with recommended best practices in tumor immunogenomics. Additionally, we utilized the IOBR package (version 2.0.0) 31, 32 for systematic investigation of TME composition and antitumor immunity, deconvoluting immune/stromal cell abundances and scoring metrics like cytolytic activity and IFN-γ response. For enhanced GSEA visualization, the GseaVis package (version 0.1.0) 33 was employed to generate interactive plots of pathway enrichments.

### TME immunological characteristics in ccRCC

As shown in Supplementary Table S2, we collected immunomodulators from the previous study 30, 34, comprising MHC, receptors, chemokines, and immunostimulants. There were seven steps that made up the cancer immune cycle: the release of cancer cell antigen (step 1), presentation of cancer antigen (step 2), initiation and activation (step 3), immune cell transport to the tumor (step 4), immune cell infiltration into the tumor (step 5), recognition of cancer cells by T lymphocytes (step 6) and killing of cancers cells (step 7) (Table S3) 35. Xu *et al.* used single sample gene set enrichment analysis (ssGSEA) to assess the activity of these processes based on the gene expression of each tumor sample 36. Based on RNA-seq data, there are numerous regularly used techniques to determine the amount of TIIC penetration in TME. Computational bias may result from the use of different TIIC methods and gene expression profile data. To eliminate bias, TIIC infiltration levels were calculated using seven different algorithms: Cibersort-ABS, MCP-counter, quantTIseq, TIMER, xCell, TIP, and TISIDB (Table S4) 36-42. Finally, the Auslander's investigation yielded 22 suppressive immunological checkpoints with therapeutic potential (Table S5) 43. Ayers *et al.* established and validated a tumor T-cell inflammation score that identifies preexisting cancer immunity and predicts clinical response to immune checkpoint blockade (ICB)^44^. Table S6 lists the 18 genes used in the T-cell inflammation score method, as well as their coefficients. The T-cell inflammation score was calculated using a weighted linear combination of the 18 gene scores in this study. We confirmed the link between HRS and TME immunological characteristics in the independent external cohort mentioned above to verify the role of HRS in maintaining cancer immunity in ccRCC.

### Cell culture and reagents

The 786-O and Caki-1 cell lines were provided by the Chinese Academy of Sciences. The 786-O cell line was cultured in RPMI 1640 medium, while the Caki-1 cell line was cultured in McCoy's 5A medium. All cells were maintained in a humidified incubator at 37 °C with 5% CO₂, with the culture media supplemented with 10% fetal bovine serum (FBS, Gibco, Thermo Fisher Scientific, USA). The PLOD2 antibody (66342-1-Ig, Proteintech) and β-Actin antibody (sc-47778, Santa Cruz) used in the experiments were purchased from commercial suppliers.

### Plasmid construction and RNA interference

To inhibit PLOD2 expression, two small interfering RNAs (siRNAs) targeting PLOD2 were used: PLOD2-siRNA1 (sense: 5'-GCCAGCUAAGAAUACAUTT-3', antisense: 5'-AUGUAUUCUAGCUCUGGCTT-3'). PLOD2-siRNA2 (sense: 5'-CAUCAUGAUAGCCGUAUAUTT-3', antisense: 5'-AUAUACUGGCTT-3'). A negative control siRNA was also included: Negative control siRNA (sense: 5'-UUCUCCGAACGUGUCACGUTT-3', antisense: 5'-UAUCGUCUGUGCAAUUAGCTT-3').

Additionally, a lentiviral vector expressing short hairpin RNA (shRNA) targeting PLOD2 was constructed and packaged by Qingke Biotechnology Co., Ltd. (Beijing, China). Cell transfection experiments were performed according to the manufacturer's instructions using Lipofectamine 3000 (Invitrogen, Carlsbad, CA, USA).

### Quantitative Real-Time PCR (qRT-PCR)

RNA extraction was performed using the RNeasy Plus Mini Kit (Qiagen, Germany) following the manufacturer's protocol. The concentration and purity of the extracted RNA were measured using the NanoDrop instrument (Implen, Germany). Subsequently, cDNA synthesis was carried out using the ReverTra Ace qPCR RT Kit (Toyobo, Japan). The qRT-PCR reactions were performed on the QuantStudio 6 Flex system (Life Technologies). The primer sequences used in the experiment were as follows: PLOD2 forward primer (PLOD2-F): 5'-CATGGACACAGGATAATGGCTG-3', PLOD2 reverse primer (PLOD2-R): 5'-AGGGGTTGGTTGCTCAATAAAAA-3'; GAPDH forward primer (GAPDH-F): 5'-TGCACCACCAACTGCTTAG-3', GAPDH reverse primer (GAPDH-R): 5'-GATGCAGGGATGATGTTC-3'. The relative gene expression levels were calculated using the 2^-ΔΔCt^ method, with GAPDH serving as the internal reference gene for normalization.

### Cell proliferation assay

Cell proliferation capacity was evaluated through colony formation assay and MTT assay. In the colony formation assay, 800 cells were seeded into 6-well plates and cultured in a 37 °C, 5% CO2 incubator for 14 days. After washing with PBS, the cells were fixed with 4% paraformaldehyde for 15 minutes and stained with 0.5% crystal violet for 10 minutes. Colonies containing more than 50 cells were counted. For the MTT assay, 3000 cells were seeded into 96-well plates with 6 replicate wells per group. Plates were retrieved daily for 5 consecutive days, and 20 µl of MTT solution (5 mg/ml, Sigma-Aldrich) was added to each well. After incubation at 37 °C for 4 hours, the supernatant was removed, and 150 µl of DMSO was added to dissolve the formazan crystals. The absorbance at 490 nm (OD value) was measured using a microplate reader, and a cell growth curve was plotted.

### Cell migration assay

Cell migration ability was assessed using the Transwell assay. Polycarbonate Transwell filters (Corning, USA) were placed in 24-well plates, with 0.2 mL of serum-free medium added to the lower chamber. A total of 5×10⁴cells were seeded in the upper chamber and incubated at 37 °C for 24 hours. After incubation, the cells were fixed with 4% paraformaldehyde and stained with 0.1% crystal violet for 10 minutes. Migrated cells were observed and counted under a microscope. Additionally, a wound healing assay was performed: transfected cells were seeded in 6-well plates, and when the cells reached 80% confluence, a vertical scratch was made using a 200 μL pipette tip. The scratched area was washed with PBS, and 2 mL of complete medium was added, followed by incubation at 37 °C for 12 hours. The wound area was observed under a microscope, and the distance between the wound edges was measured to calculate the wound healing rate.

### Xenograft tumor experiment

The experiment strictly adhered to the National Institutes of Health Guidelines for the Use of Laboratory Animals and was approved by the Institutional Animal Care and Use Committee of Wuhan University. Three-week-old male BALB/cnu mice were acclimatized for 7 days in a specific pathogen-free (SPF) environment and then randomly divided into 2 groups (n = 6). Caki-1 cells infected with LV-control (NC group) or LV-shRNA (sh-PLOD2 group) lentivirus (5×10⁶ cells in 100 µl of serum-free medium) were subcutaneously injected into the dorsal flank of each mouse. Tumor length (L) and width (W) were measured every 3 days using a caliper, and tumor volume (mm³) was calculated using the formula V = (L × W²)/2. After continuous monitoring for 45 days, the mice were euthanized, and tumor weight was measured. Tumor tissues were collected for hematoxylin and eosin (H&E) staining and immunohistochemical analysis.

### Immunofluorescence staining

A solution of cold PBS was used to wash cells inoculated on coverslips before they were fixed for 30 minutes with 4% PFA and treated with 0.1%Triton X100 for 15 minutes. Cells were blocked with 5% BSA for 30 minutes and treated with the primary antibody for 2 hours. A Cy3-labeled secondary antibody is applied to the cells for an hour at room temperature after they have been washed with PBS. In order to examine the immunofluorescence staining, the nuclei were labeled with DAPI (Olympus, Japan) and observed under a fluorescent microscope.

### Statistical analysis

Statistical significance for comparisons between two or more variables was assessed using Student's t-test or one-way analysis of variance (ANOVA). Overall survival (OS) differences were analyzed with Kaplan-Meier survival curves, and the log-rank test was conducted using the R survminer package 33. Spearman correlation analysis was used to determine the correlation distance between parameters, while two-sided Fisher's exact test evaluated differences in immune checkpoint inhibitor (ICI) responses. Independent prognostic factors were identified through univariate and multivariate Cox proportional hazards models and visualized with R forestplot package. Somatic mutations were visualized as waterfall plots using R maftools and complexheatmap packages, and subject operating characteristic (ROC) curves were plotted with R survivalROC package, with the area under the curve (AUC) used to assess diagnostic accuracy.

## Results

### High hypoxia level indicated worse survival in ccRCC patients

**Figure 1:**
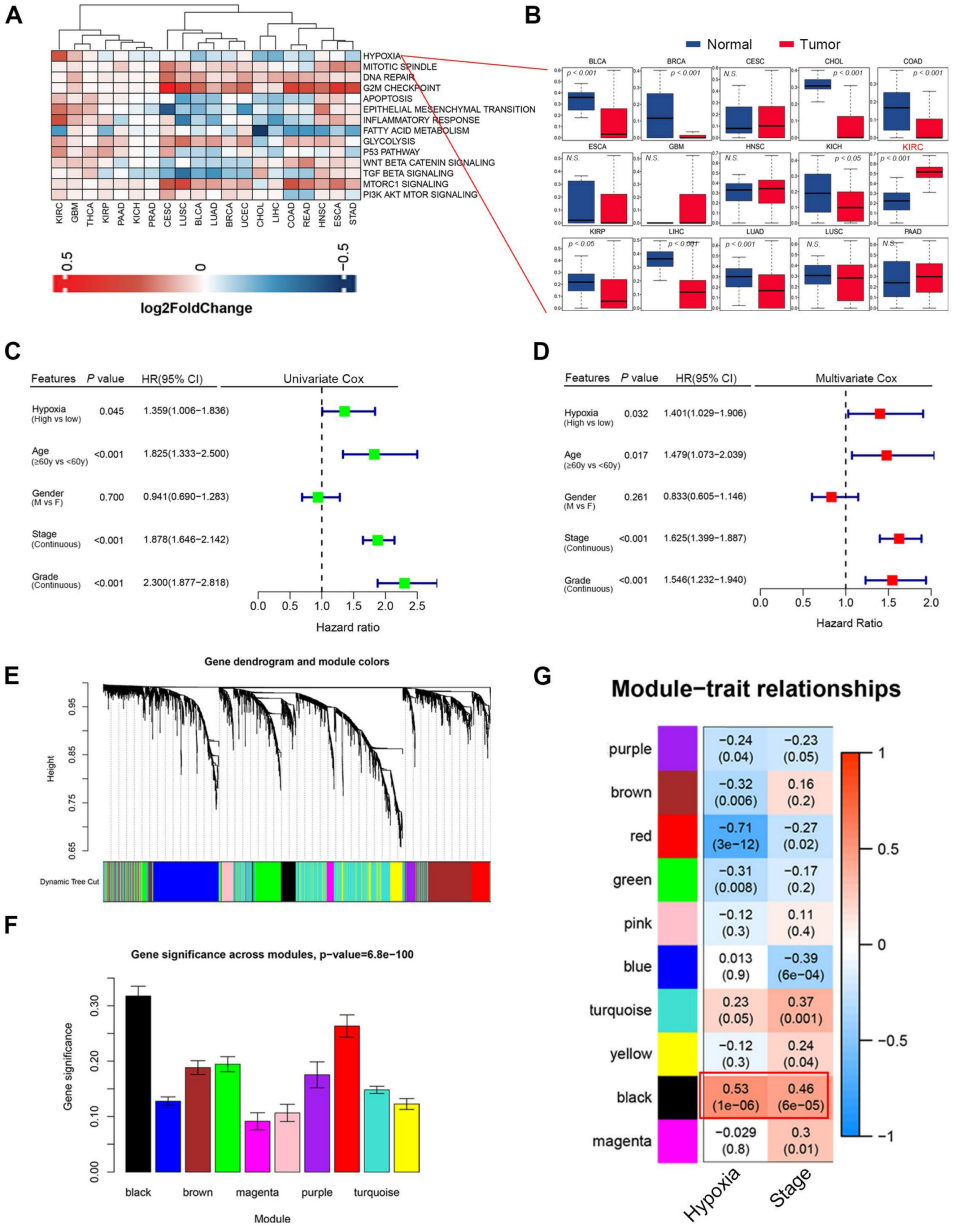
(A) Cancer hallmark enrichment scores across 20 cancer types. Red represents hallmark scores that are upregulated, and blue represents those that are downregulated. (B) Boxplots showing the distribution of hypoxia level between tumors and normal samples. (C) Univariate Cox regression analysis indicated that hypoxia was the significant variable for overall survival. (D) Hypoxia was the significant variable in the multivariate Cox regression analysis among clinicopathological features (P = 0.032). (E) In WGCNA analysis, using the 1-TOM dissimilarity measure, a dendrogram of gene clusters is shown. (F) The distribution of average gene significance and errors in the modules associated with hypoxia levels of ccRCC is shown. (G) Heatmap showing the correlation between modules and enrichment scores of hypoxia level and tumor stages.

We found that hypoxia levels in clear cell renal cell carcinoma (ccRCC) was significantly higher than those in normal kidney tissues (Fig. 1A-B, Fig. S1A). The result was further supported by the results from the ccRCC microarray datasets GSE40355 and GSE53757 (Fig. S1B-C). Univariate and Multivariate Cox regression analysis identified hypoxia as a significant risk factor for clinical outcomes, standing out among various cancer-related factors (*P* = 0.032, HR = 1.401, Fig. 1C-D). To identify the hypoxia-induced biomarkers in ccRCC, we constructed a co-expression network using transcriptome data and hypoxia level. A soft threshold power of 4 was applied to create a topological matrix with scale-free characteristics (R² = 0.85; Fig. 1E-F, Fig. S2A-C). This analysis identified 10 distinct gene modules (Fig. 1E-F). The correlation between module features, hypoxia scores, and clinical characteristics was visualized using a heatmap (Fig. 1G). Among the modules, the black modules showed the strongest positive correlation with hypoxia. The black module also demonstrated a higher association with tumor stage (r = 0.46; Fig. 1G). Hypoxia was also found to be significantly and positively correlated with EMT and immune response (Fig. S2D-E). These results suggest that hypoxia contributes to alterations in the tumor microenvironment, promoting EMT and immune evasion.

### Development and validation of a Hypoxia Risk Score (HRS)

**Figure 2:**
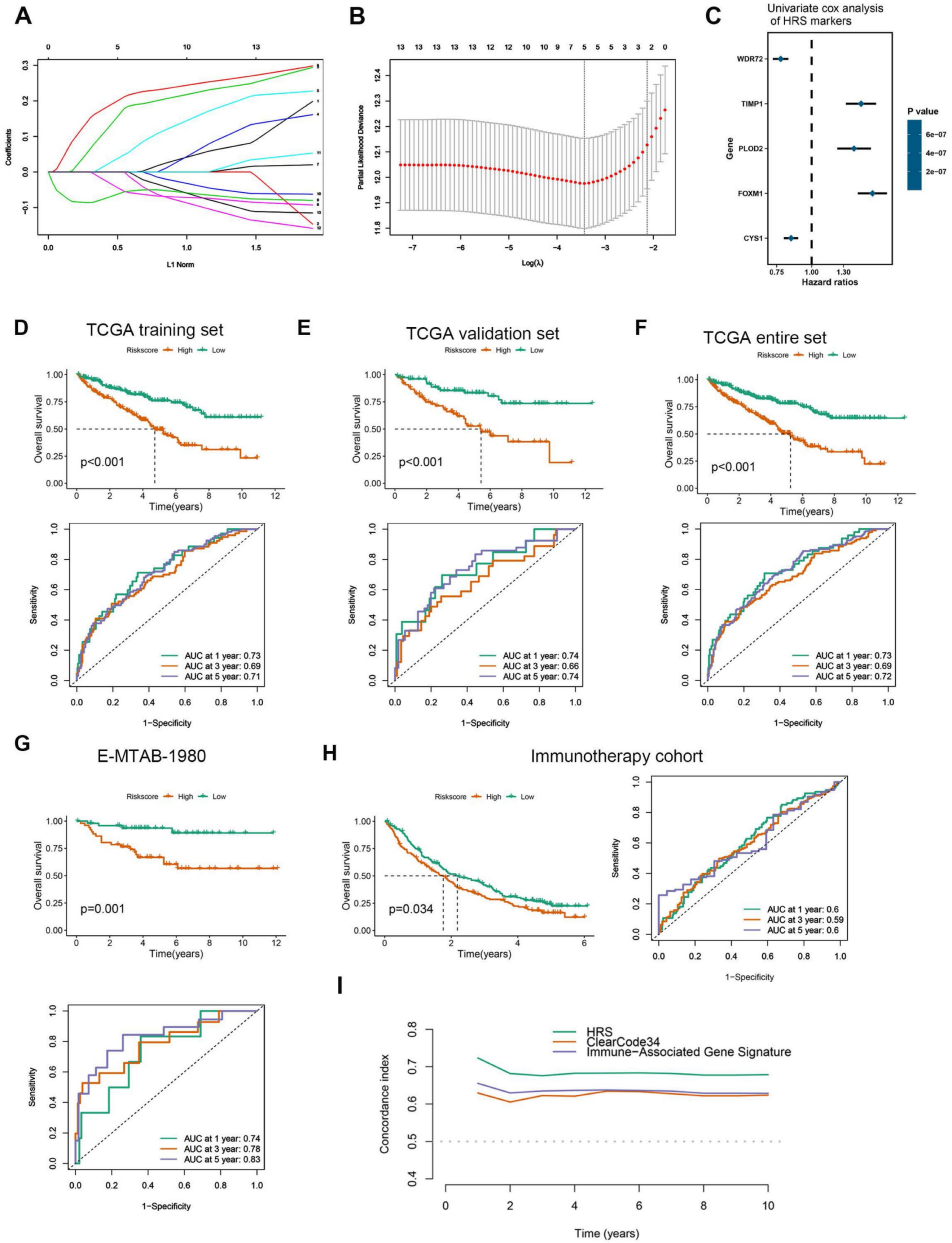
Based on LASSO Cox regression, a hypoxia risk score (HRS) model was developed. (A) LASSO coefficient profiles of genes associated with disease prognosis in TCGA training cohorts (n=363). Using the log (Lambda) sequence, the coefficient profile plot was developed. (B)In the LASSO regression model, cross-validation for parameter selection is done by using minimum criteria. Using the minimum criteria, two vertical dotted lines were plotted at the optimal values. (C) In univariate cox analysis, a forest plot representing the HRS gene expression profile was generated using the five genes with the best discriminative capability. (D) Development of HRS for TCGA training set and survivability predicting accuracy for HRS. (E-F) Evaluation of the HRS in the TCGA validation and entire sets. (G-H) In this paper, we validated the HRS expression profiles using two independent external datasets: E-MTAB-1980 (n = 101), and immunotherapy cohort (n = 311). (I) Concordance index was caculated to compare the HRS with biomarker-based prognostic models including ClearCode34 (ccRCC molecular subtyping model) and immune gene-based signatures. HRS achieved a significantly higher concordance index for overall survival prediction compared to ClearCode34 and immune gene signature.

The hub genes with the highest module membership (MM) and gene significance (GS) were identified from the co-expression network. These include FOXM1, TGFBI, TIMP1, WDR72, PLOD2, C1S, GPAT3, CYS1, C1R, OSMR, PTPN3, TRPM3, GPX8, and LHFPL2. Given that HIF1A is a central transcription factor in hypoxia, we observed a significant correlation between HIF1A expression and the expression levels of these 14 core genes (Fig. S3). To quantify an individual's hypoxia risk in ccRCC, we developed a hypoxia risk score (HRS). In the TCGA ccRCC cohort, we applied LASSO Cox regression analysis on the 14 hypoxia-related genes. From this analysis, the five most predictive genes were selected using the smallest λ value (0.0214) to generate the HRS in the TCGA training cohort (Fig. 2A, B). The coefficients for these five genes are provided (Fig. 2C, Table S7). Patients in the TCGA training cohort were stratified into low and high HRS groups. A significant difference in overall survival (OS) was observed between these groups (Fig. 2D). The HRS demonstrated strong predictive power for 1-, 3-, and 5-year OS, with accuracies of 0.73, 0.69, and 0.71, respectively (Fig. 2D). We further validated the HRS's predictive capability for OS in the TCGA validation cohort (Fig. 2E, F). To evaluate the generalizability of the HRS, we applied it to two independent ccRCC datasets. In the immunotherapy cohort, patients in the high-risk group exhibited significantly worse OS compared to those in the low-risk group (Fig. 2G), with prediction accuracies for 1-, 3-, and 5-year OS of 0.74, 0.78, and 0.83, respectively (Fig. 2G). More importantly, for ccRCC patients receiving immunotherapy, higher HRS was associated with worse OS, suggesting that the patients with higher HRS derived less benefit from immunotherapy (Fig. 2H). Therefore, this risk model is helpful to predict the immunotherapy response in ccRCC patients.

Compared HRS with biomarker-based prognostic models applicable to our datasets: ClearCode34^45^ (ccRCC molecular subtyping model) and immune gene-based signatures 22 (e.g., 5-gene inflammatory score). HRS achieved a significantly higher concordance index for overall survival prediction compared to ClearCode34 and immune gene signature (Fig. 2I and Fig. S7). Time-dependent ROC analysis confirmed HRS's superior predictive accuracy for 1/3/5-year survival (AUCs: 0.73/0.69/0.72) over comparator models. These results demonstrate that HRS provides independent prognostic value complementary to existing biomarker-driven approaches.

### HRS predicts the immune landscape in the tumor microenvironment

**Figure 3:**
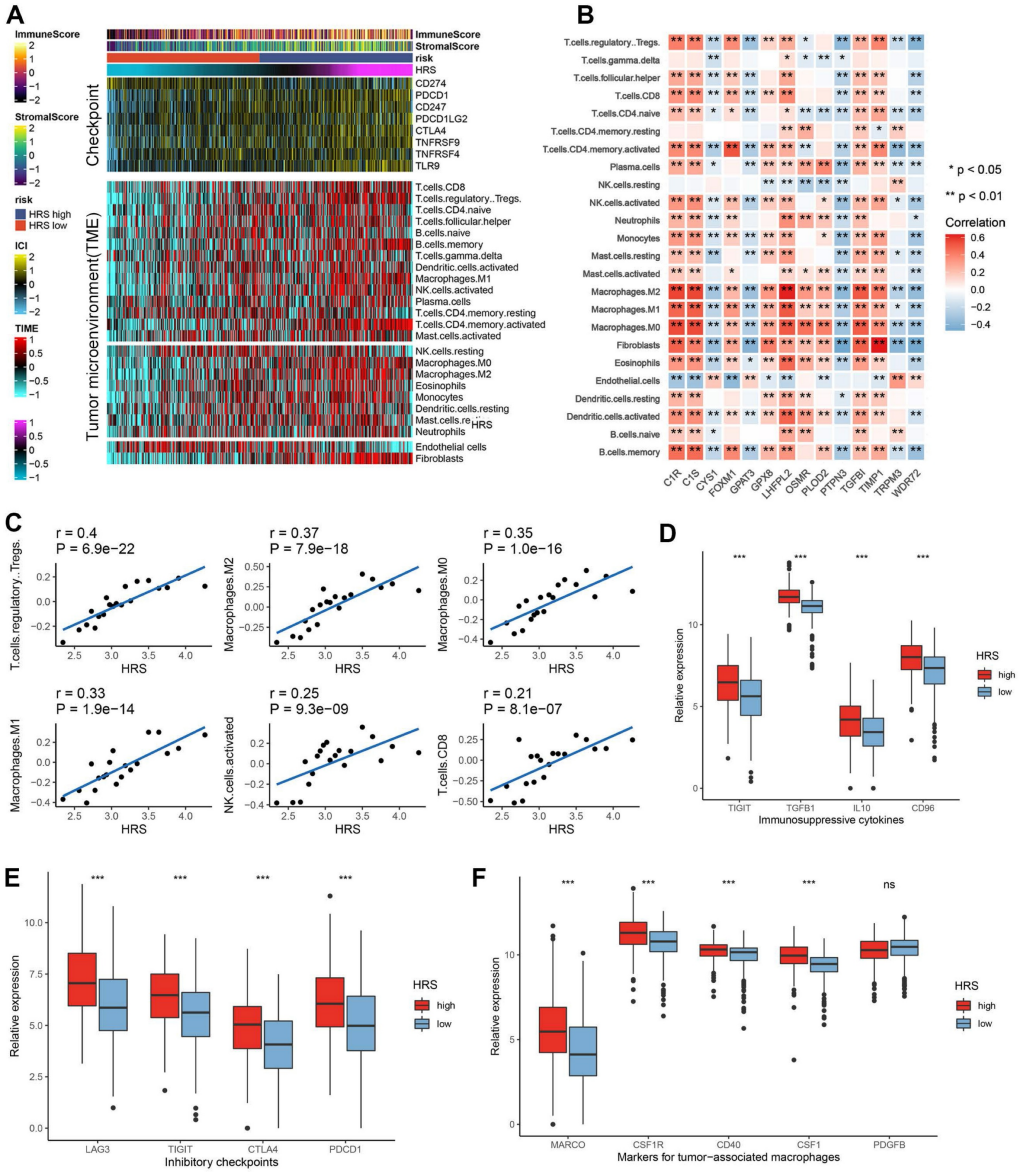
(A)The top panel of the heatmap depicts the expression levels of genes involved in immune checkpoint targets, while the bottom panel shows the enrichment levels of 24 microenvironment cell types. Annotations at the top of the heatmap include immune enrichment scores, stromal enrichment scores and hypoxia risk scores (HRS). Analysis of the relationship between 14 hypoxia signatures and immune cell infiltration. (C) Correlation of HRS with Treg, Macrophage and CD8+ T cell infiltration. (E) Bar plot showing immunosuppressive cytokines in HRS low and high groups. (F) Bar plot showing expression of inhibitory checkpoints in HRS low and high groups. (G)Bar plot showing expression of tumor associated macrophages' makers in HRS low and high groups.

To explain the mechanism of the differences in immunotherapy responses among different HRS groups, we performed a more in-depth analysis. We found that the tumor microenvironment landscape had a dramatic difference in immune infiltration between high and low HRS groups (Fig. 3A). We found that ccRCC patients with high HRS showed higher immune cell infiltration, especially CD4+ and CD8+ T cells, and Treg (Fig. 3A). Using IOBR, high-HRS tumors exhibited elevated immunosuppressive signatures (e.g., higher MDSC and Treg infiltration) (Fig. S9A-C). GseaVis analysis confirmed enriched metabolic and immune evasion (TGF-β signaling) terms in high-HRS group (Fig. S9D). Besides, the expression levels of gene sets in HRS were mostly positively correlated with the infiltration levels of immune cells (Fig. 3B-C). At the same time, inhibitory inflammatory factors, inhibitory immune checkpoints, and tumor-associated macrophages were all upregulated in ccRCC patients with high HRS (Fig. 3D-F). These results suggest that the reason why the high HRS group has a poor response to immunotherapy may be related to tumor immunosuppression and immune escape.

**Figure 4:**
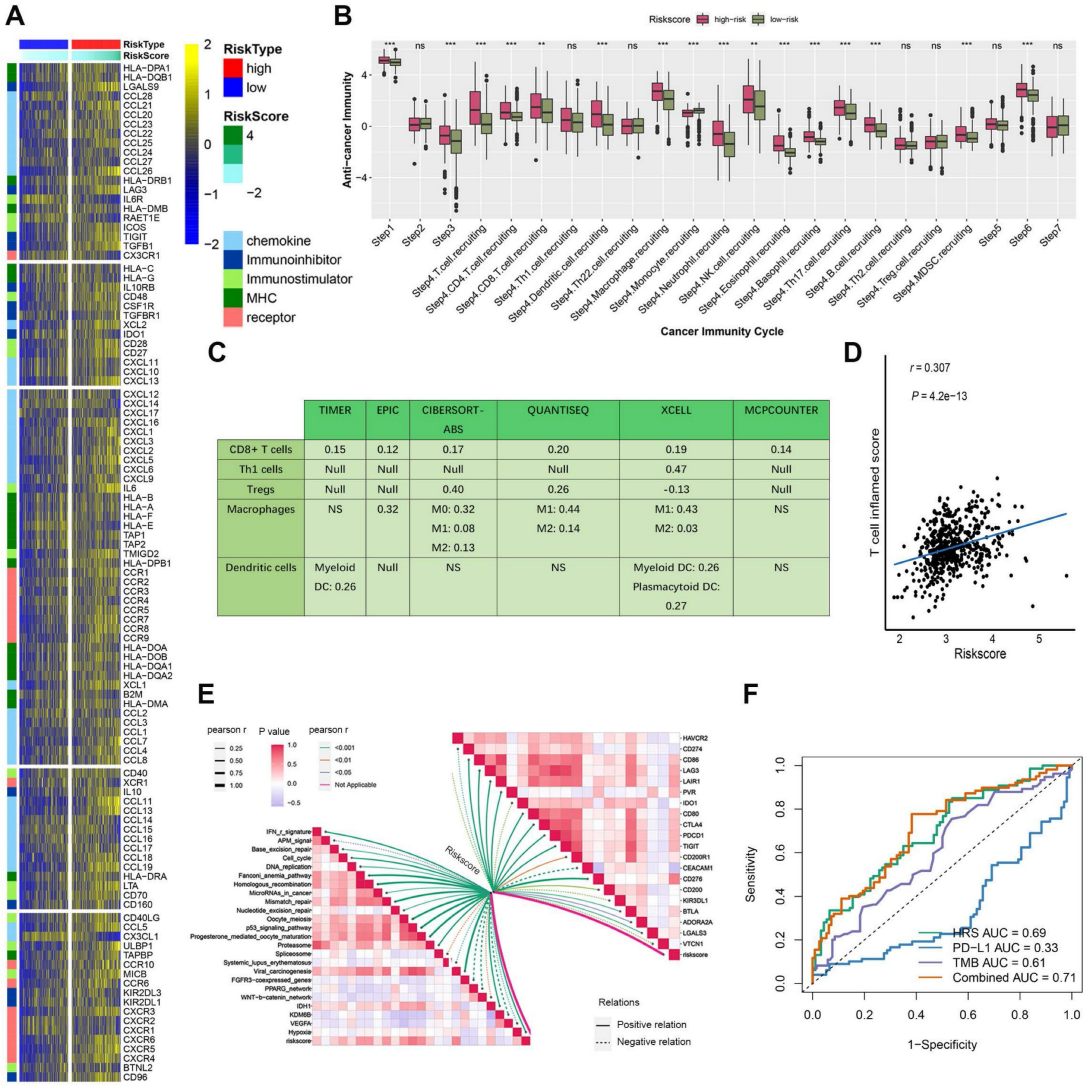
High HRS in CCRCC patients creates an inflamed TME. (A) Differences in the expression of 122 immunomodulators (chemokines, receptors, MHCs, and immunostimulators) between high-risk and low-risk groups in ccRCC. (B) Differences between high- and low-risk groups at each step of the cancer immunity cycle. (C) Using seven independent algorithms, we calculated the correlation between HRS and the infiltration of CD8+ T cells, macrophages, Th1 cells, and dendritic cells. (D)The correlation between HRS and the pan-cancer T cell inflamed score. (E) Correlation between HRS and enrichment scores of immunotherapy-predicted pathways and immune checkpoints. (F) AUC values show that the combination of HRS with traditional biomarkers (TMB, PD-L1) improves predictive accuracy for ICB response. (*P < 0.05; **P < 0.01; ***P < 0.001).

Furthermore, the hypoxia risk score (HRS) was found to be positively associated with various immunomodulators (Fig. 4A, Table S2). Notably, several MHC molecules were elevated in the high-risk group, indicating an enhanced capacity for antigen presentation and processing (Fig. 4A-B). In addition, two key chemokines, CXCL9 and CCR3, which are known to promote CD8+ T cell recruitment into the tumor microenvironment (TME), were upregulated in the high-risk group. Chemokines and their receptors, which influence the recruitment of immune cells such as CD8+ T cells, TH17 cells, and antigen-presenting cells, were also implicated (Fig. 4A). However, due to the complexity and polymorphism of the chemokine system, the relationship between HRS and individual chemokines may not fully explain the overall immunological role of HRS in the TME.

The activity of the cancer immune cycle reflects the combined functions of the chemokine system and other immunomodulators. In the high-risk group, most steps in the immune cycle were upregulated, including the release of cancer cell antigens (step 1), priming and activation (step 3), and trafficking of immune cells to the tumor (step 4), such as the recruitment of CD8+ T cells, macrophages, NK cells, dendritic cells (DCs), and TH17 cells (Fig. 4B). This increased activity is likely to enhance the infiltration of effector tumor-infiltrating immune cells (TIICs) in the TME. The infiltration levels of TIICs were calculated using seven independent algorithms (Fig. 4C, Table S4). Consistent with previous findings, HRS was positively correlated with CD8+ T cells, Tregs, macrophages, and dendritic cells across different algorithms (Fig. 4C).

Moreover, HRS showed a significant positive correlation with the pan-cancer T cell inflamed score (R = 0.307, *P* = 4.2e-13; Fig. 4D). Inflammatory TMEs are known to express high levels of immune checkpoint inhibitors, such as PD-L1/PD-1. In this study, HRS was positively correlated with several immune checkpoint markers, including PD-1, CTLA-4, LAG-3, CD276, and TIGIT (Fig. 4E, Table S5). These findings suggest that ccRCC patients with higher HRS may respond more favorably to immune checkpoint blockade (ICB), as HRS defines an inflamed TME. To further compare the predictive power of HRS with traditional immunotherapy biomarkers, we performed ROC analysis using PD-L1 expression, TMB, and their combination with HRS. The combination model (TMB + HRS + immune gene expression) achieved the highest AUC (0.71), significantly outperforming TMB alone (AUC = 0.61) or PD-L1 expression alone (AUC = 0.33), suggesting that HRS can effectively complement existing biomarkers (Fig. 4F).

To further validate the role of HRS in predicting immune phenotypes and therapeutic opportunities, we applied an independent validation dataset. Consistent with previous findings, HRS was positively associated with a broad range of immunomodulators, and it was also positively correlated with the enrichment score of the anticancer immune cycle (Fig. S4A, E). Similarly, HRS was associated with increased infiltration levels of TIICs, including CD8+ T cells, Tregs, and dendritic cells (Fig. S4C). Furthermore, HRS was positively correlated with the enrichment scores of immune checkpoints, the T cell inflamed score (TIS), and ICB response-related features (Fig. S4B). In the validation cohort, patients in the high-risk group displayed a distinct phenotype characterized by T cell infiltration (Fig. S4D).

### Association of HRS with Tumor Mutational Burden (TMB)

**Figure 5:**
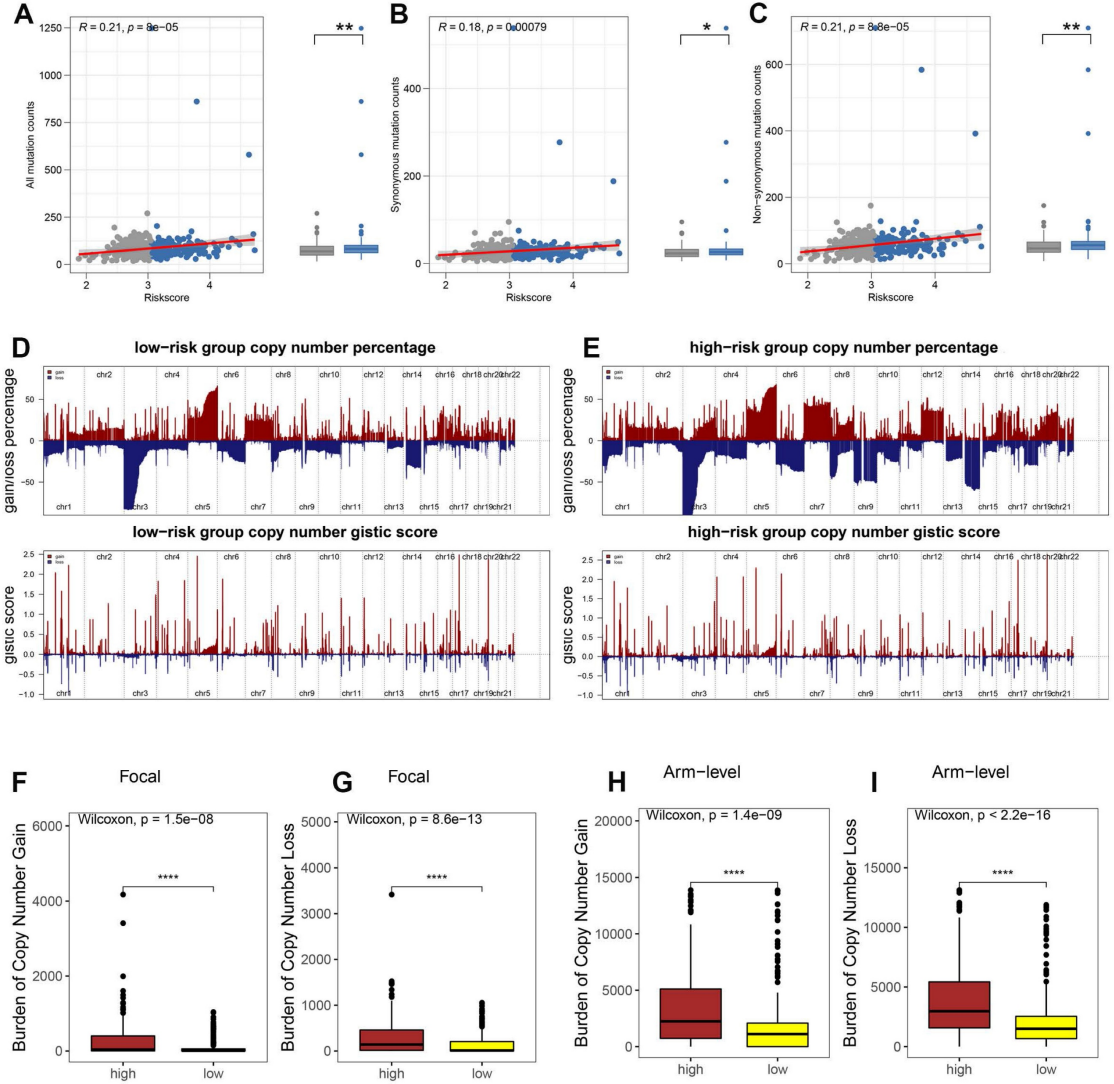
HRS was associated with tumor mutation status. The association between all mutation counts (A), synonymous mutation counts (B), and non-synonymous mutation counts (C), and their distribution in the low- and high-risk groups. (D-E) Comparison of the copy number variance in high- and low-risk groups of ccRCC. Above is a graph showing the frequency of the gains and losses. Below are plots showing the cytoband with focal amplification and deletion generated by the GISTIC_2.0 software. The horizontal axis represents the q value of each locus. (F-I) Distribution of focal and broad copy number variations between groups at low and high risk.

Tumor immune escape is driven by somatic copy number alterations (SCNAs) and mutations. To investigate the role of the hypoxia risk score (HRS) in this mechanism in ccRCC, we analyzed somatic mutations in the TCGA database. Our findings revealed that the high HRS group had a greater number of somatic mutations, both non-synonymous and synonymous, compared to the low HRS group (Fig. 5A-C). Emerging evidence suggests that a high burden of copy number loss is correlated with resistance to anti-PD-1 and anti-CTLA-4 therapies, indicating that copy number loss is closely linked to tumor immune evasion 46. Thus, we explored the differences in SCNAs between the immune groups. In both the high-risk and low-risk groups, genomic amplifications and deletions were observed (Fig. 5D-E), notably the amplification of chromosome arms 5p and 7p and the loss of 3p and 14q. The lower panels in Figures 5D and E display the distribution of SCNAs across all chromosomes, while the upper panels show the gains and losses of SCNAs. High-risk patients exhibited a significantly greater burden of both focal and arm-level SCNAs than low-risk patients (P < 0.0001, Fig. 5F-I). Table S8 provides the SCNA burden for each sample in the TCGA-KIRC cohort. These results suggest that, under hypoxic conditions, the recruitment of inhibitory immune cells and immunosuppressive factors, along with alterations in the tumor microenvironment, allow high-risk ccRCC patients to evade immune surveillance, leading to immune escape. Additionally, the high HRS group showed a higher tumor mutation burden (TMB), which is often associated with increased immunogenicity and improved responses to immunotherapy. While TMB's predictive value in ccRCC remains debated, our findings suggest that the combination of high HRS and elevated TMB may provide a more robust predictive framework than either parameter alone. This further supports the use of HRS as a predictive marker for immune checkpoint blockade (ICB) response. By identifying patients with both high HRS and elevated TMB, clinicians may be able to better stratify ccRCC patients who are more likely to benefit from ICB therapy, optimizing personalized treatment strategies.

### PLOD2 as a biomarker of hypoxia-driven tumor progression and immune response

**Figure 6:**
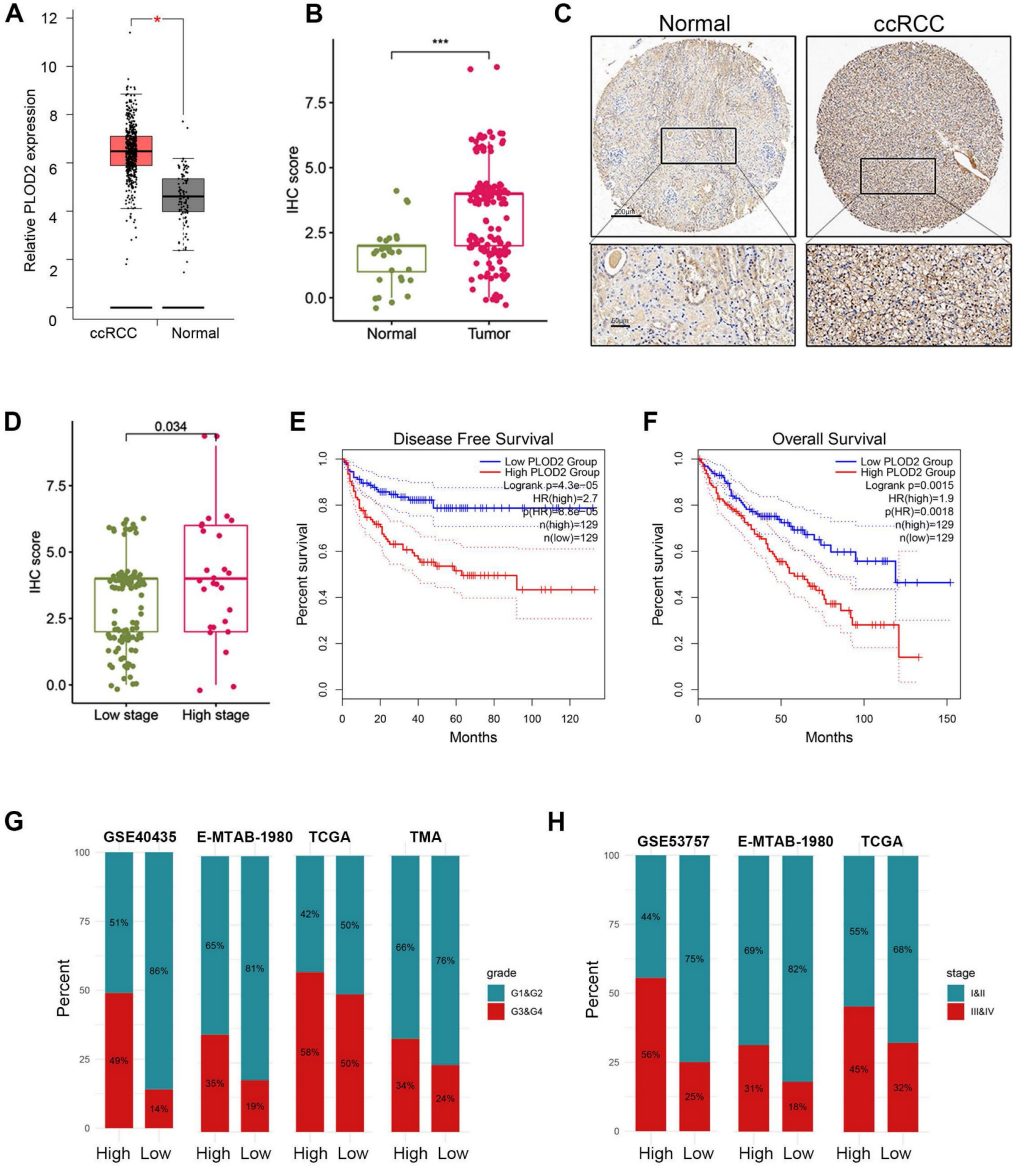
PLOD2 was highly expressed in ccRCC. (A) Boxplot showed the PLOD2 mRNA expression in ccRCC and normal kidney from TCGA database. (B) PLOD2 staining was performed on 150 ccRCC tissues and 30 adjacent normal tissues. (C) Immunostaining images of PLOD2 expression in the tissue microarray of ccRCC. The scale bar is 200mm. (D) The staining score for PLOD2 in ccRCC with low stage (I) and high stage (II-IV). (E) Patients with ccRCC with high PLOD2 expression have significantly reduced overall survival in TCGA database. (F) Patients with ccRCC with high PLOD2 expression have significantly reduced disease-free survival in TCGA database. (G) In TCGA, GSE40435, E-MTAB-1980 and tissue microarray (TMA) cohorts, the distribution of G1&G2 and G3&G4 tumors was compared between high- and low-PLOD2 groups. (H) In the TCGA combined GSE53757 and E-MTAB-1980 cohort, distribution of tumors stage I/II and III/IV will be compared between the high- and low-PLOD2 groups. (C) (D) Analysis of microarray data of GSE53757 shows a correlation between PLOD2 expression and ccRCC stage.

Previous studies have demonstrated that PLOD2 is induced under hypoxic conditions and acts as a target gene of HIF1A^47^. Downregulation of PLOD2 has been shown to inhibit proliferation and metastasis in clear cell renal cell carcinoma (ccRCC)^47^. However, the association between PLOD2 and immune infiltration or immunotherapy response in ccRCC has not been explored. Given its role as a key component of the hypoxia risk score (HRS), we investigated the relationship between PLOD2 and the immune microenvironment, as well as its influence on immunotherapy outcomes in ccRCC. We confirmed that PLOD2 mRNA expression was significantly elevated in higher tumor stages (III and IV) and higher Fuhrman grades (G3 and G4) compared to lower stages (I and II) and grades (G1 and G2) (Fig. 6A- B). Across different cohorts, PLOD2 expression showed a positive correlation with tumor stage and Fuhrman grade in ccRCC (Fig. 6C-E). Furthermore, immunohistochemistry (IHC) staining of 150 ccRCC tissue microarrays (TMAs) confirmed higher PLOD2 protein levels in advanced-stage tumors (Fig. 6F-H). Survival analysis using ccRCC tissue microarray data revealed that patients with elevated PLOD2 expression had significantly shorter overall survival (OS) times (Fig. 6I). We identified 142 differentially expressed genes (DEGs) associated with high PLOD2 expression, which were predominantly linked to immune-related pathways (Fig. S5A). Gene Ontology analysis highlighted processes such as the acute phase response and extracellular matrix organization (Fig. S5B). In a cohort of 156 metastatic ccRCC patients treated with nivolumab (an anti-PD-1 therapy), high PLOD2 expression was associated with increased sensitivity to immune checkpoint blockade (ICB) (Fig. S5C). Furthermore, we observed frequent PBRM1 mutations in both high and low PLOD2 groups, with a higher distribution of PBRM1 mutations in the high-expression group (Fig. S5F- G). The strong correlation between hypoxic status and PBRM1 mutations (*P* < 0.01) in ccRCC patients suggests a link between these factors in tumor progression. Given that PBRM1 loss-of-function (LOF) mutations have been linked to survival and ICB response in ccRCC^48^, ^49^, we examined the relationship between PBRM1 LOF and PLOD2 expression. Our results revealed a higher incidence of PBRM1 LOF mutations in the high PLOD2 group (81 vs. 58,* P* < 0.01, Fig. S5G). Combining PBRM1 mutation status with PLOD2 expression significantly improved risk stratification (Fig. S5H), suggesting that PLOD2 could serve as both a marker of aggressive disease and a predictor of suboptimal immunotherapy outcomes. These findings highlight the potential of PLOD2 as a therapeutic target in ccRCC, particularly in high-risk patients with poor prognosis. Targeting PLOD2 may offer a novel strategy to enhance therapeutic responses, particularly for those with elevated PBRM1 mutations or hypoxic tumor microenvironments.

### PLOD2 promotes proliferation and migration of clear cell renal cell carcinoma *in vitro* and *in vivo*

**Figure 7:**
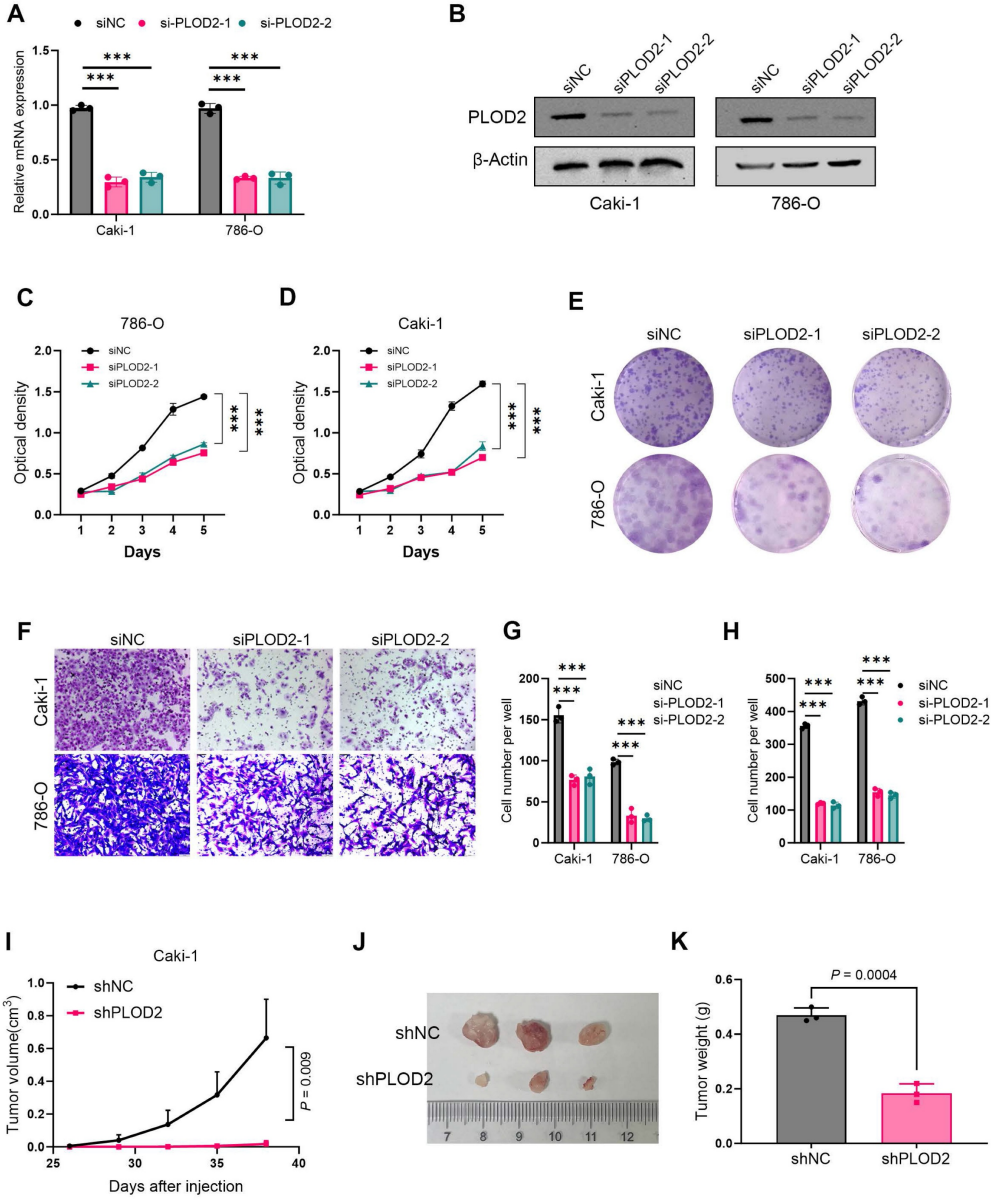
PLOD2 knockdown inhibits proliferation and migration of clear cell renal cell carcinoma cells. (A) The knockdown efficiency of two PLOD2-specific siRNAs in 786-O and Caki-1 cells was evaluated by qRT-PCR analysis. (B) Immunoblot analysis further validated the knockdown efficiency of siPLOD2-1 and siPLOD2-2 in 786-O and Caki-1 cells. (C-D) MTT assay results demonstrated that PLOD2 knockdown significantly reduced the proliferation viability of 786-O and Caki-1 cells. (E) Colony formation assays and statistical analysis revealed that PLOD2 knockdown significantly inhibited the colony-forming ability of 786-O and Caki-1 cells. (F) Transwell assays confirmed that PLOD2 knockdown significantly suppressed the migration ability of 786-O and Caki-1 cells. (G-H) Quantitative statistical analysis of Transwell and wound healing assays. (I) In the xenograft tumor model, PLOD2 knockdown significantly inhibited tumor growth (n=3 per group). (J) Representative images of tumors from the xenograft mouse model. (K) PLOD2 knockdown significantly reduced the tumor weight in the xenograft model (n=3 per group). Statistical significance was determined using two-tailed t-tests. Error bars represent the standard deviation of three independent experiments. ***p < 0.001, **p < 0.01, *p < 0.05.

To investigate the role of PLOD2 in the proliferation of clear cell renal cell carcinoma (ccRCC), we knocked down PLOD2 in 786-O and Caki-1 cells (Fig. 7A, B). The results showed that PLOD2 knockdown significantly inhibited cell proliferation viability (Fig. 7C, D) and colony formation ability (Fig. 7E, G). To further validate the effect of PLOD2 on cell migration, Transwell and wound healing assays were performed using 786-O and Caki-1 cells. The results demonstrated that PLOD2 knockdown significantly reduced the migration capacity of ccRCC cells (Fig. 7F, H). To explore the role of PLOD2 in tumor growth *in vivo*, we established a xenograft mouse model. PLOD2 was knocked down in Caki-1 cells using lentivirus-mediated shRNA, and Caki-1 LV-control cells (NC group) and Caki-1 LV-shPLOD2 cells were injected into BALB/c nude mice, respectively. The results showed that tumor growth in the shPLOD2 group was significantly slower than that in the NC group (Fig. 7I-J), and the tumor weight was significantly reduced (Fig. 7K). In conclusion, PLOD2 knockdown inhibits the proliferation and migration of ccRCC cells both *in vitro* and *in vivo*.

## Discussion

Our study explores of the intricate relationship between hypoxia, tumor immune microenvironment, and immunotherapeutic responses in clear cell renal cell carcinoma (ccRCC). By developing a novel hypoxia risk score (HRS), we uncovered critical insights that transcend traditional understanding of tumor progression and immune dynamics. A key contribution of our research lies in the comprehensive characterization of how hypoxia fundamentally reshapes the tumor immune microenvironment (TME). Unlike previous studies that considered hypoxia as a passive oncogenic phenomenon, our findings reveal it as an active modulator of immune cell recruitment and functional activation. Mechanistically, we demonstrated that high hypoxia risk scores are not merely correlative but mechanistically linked to profound immune landscape transformations 41, 46. These finding challenges existing paradigms by illustrating hypoxia as a potential orchestrator of immune cell dynamics rather than a simple passive environmental factor 50. Specifically, HIF-1α-driven glycolysis reprograms the TME, enhancing immunosuppressive signaling and justifying the mechanistic basis of our HRS model 51, 52.

Our investigation also reveals a complex interplay between somatic copy number alterations (SCNAs), genetic variations, and immune system interactions. Genomic heterogeneity within ccRCC, particularly the variations in SCNA distribution, offers insights into tumor immune evasion mechanisms 53. We demonstrated that high SCNA burden are associated with reduced immunogenicity and potentially compromised immunotherapeutic responses. Somatic variations and copy number changes can lead to immune invasion 54. Moreover, SCNAs and somatic variants influence responses to tumor immunotherapy, and patients with high SCNA have a poorer response to immunotherapy 55. Genetic testing for SCNA and somatic variants revealed significant differences in immune cell infiltration between high-risk and low-risk individuals who responded to immune checkpoint blockade drugs. In high-risk groups, the expression of PD-1 protein in the immune examination site is significantly increased, and resistance to PD-1 inhibitors is also increased. Our study highlights that while conventional biomarkers like TMB and PD-L1 expression have limited predictive value in ccRCC, the integration of HRS markedly improves stratification performance. HRS captures both immune-activating (e.g., high TMB) and immune-suppressive features (e.g., hypoxia-driven suppression), thus offering a more comprehensive measure of tumor immune phenotype. This integrative approach is consistent with recent studies emphasizing multidimensional biomarkers in optimizing ICB stratification 22.

Our findings show that PLOD2 is not merely a passive gene but an active participant in tumor progression. Its correlation with neutrophil accumulation and potential role in modulating immune checkpoint sensitivity opens new avenues for personalized therapeutic strategies. The strong association between PLOD2 expression, PBRM1 mutations, and hypoxic status provides a multilayered understanding of ccRCC's molecular complexity. This multilayered approach distinguishes our study from previous research by offering a more nuanced perspective on tumor heterogeneity 56. These findings suggest that PLOD2 could serve as both a marker of aggressive disease and a predictor of suboptimal immunotherapy outcomes. Mechanistically, hypoxia-induced PLOD2 stabilizes collagen via lysyl hydroxylase, promoting ECM stiffening and EMT (EGFR/AKT activation), driving progression 47. Paradoxically, this enhances ICB sensitivity by neoantigen release (ECM remodeling-apoptosis) and T-cell infiltration, creating an inflamed TME 56.

Although our study demonstrates HRS's predictive value for ICB response, translating it into clinical practice requires standardized protocols. We propose a workflow: (1) Pre-treatment biopsy RNA-seq or NanoString nCounter for HRS calculation (threshold: median = 3.05 from TCGA); (2) Composite scoring with TMB (> 10 mut/Mb) and PD-L1 (CPS ≥ 1); (3) Risk-stratified guidelines—low-HRS for ICB monotherapy; high-HRS for combinations (e.g., ICB + axitinib to alleviate hypoxia). Validation in trials such as NCT04586231 and a web-calculator (e.g., via MSK-IMPACT) will enhance applicability across ethnicities. This positions HRS as a decision-support tool for personalized ccRCC management.

Nonetheless, our study has several limitations that warrant consideration. The immunotherapy cohort (n = 311) had limited metadata, potentially affecting generalizability; HRS's C-index = 0.72 outperforms ClearCode34 (0.65) and TIS (0.68) in TCGA/GEO but requires multi-ethnic validation (e.g., Asian cohorts underrepresented in TCGA). Retrospective design limits causality inference, and regimen-specific data is absent. Future directions include: (1) Prospective RCTs stratifying by HRS+PLOD2 IHC; (2) Spatial transcriptomics for hypoxia-TME gradients; (3) AI-enhanced HRS with scRNA-seq integration.

In summary, by integrating hypoxia-induced gene signatures, immune cell dynamics, and genomic variations, our hypoxia risk score model emerges as a powerful prognostic and predictive tool. It transcends traditional single-dimensional approaches, offering a comprehensive framework for understanding ccRCC's biological intricacies. Future research should focus on validating these findings in larger, diverse patient cohorts and exploring potential therapeutic interventions targeting the identified molecular mechanisms. Our study lays a foundation for personalized immunotherapeutic strategies in renal cell carcinoma.

## Supplementary Material

Supplementary figures.

Supplementary tables.

## Abbreviations

WGCNA: Weighted Gene Co-expression Network Analysis
ccRCC: Clear Cell Renal Cell Carcinoma
EMT: Epithelial-Mesenchymal Transition
TCGA: The Cancer Genome Atlas
KIRC: Kidney Renal Clear Cell Carcinoma
LASSO: Least Absolute Shrinkage and Selection Operator
ICI: Immune Checkpoint Inhibitor
TIICs: Tumor-infiltrating immune cells
TIME: Tumor Immune Microenvironment
TME: Tumor Microenvironment
HRS: Hypoxia risk score
ICB: Immune Checkpoint Blockade
GO: Gene Ontology
BP: Biological Process
ssGSEA: Single-sample Gene Set Enrichment Analysis
FDR: False Discovery Rate
FC: Fold Change
TOM: Topological Overlap Matrix
OS: Overall Survival
CNV: Copy Number Variation
