# Supplementary material for: Hypoxia-Driven Immune Escape in Clear Cell Renal Cell Carcinoma: A Prognostic Model and Dual-Functional Biomarker PLOD2 for Immunotherapy Stratification
Source: J Cancer. 2026 Jan 1;17(2):217–34. doi: 10.7150/jca.114151 (PMC12825148; doi:10.7150/jca.114151)
Supplement: Supplementary file 1 — Supplementary figures. [file jcav17p0217s1.pdf]

Hypoxia-Driven Immune Escape in Clear Cell Renal Cell Carcinoma: A Prognostic Model and Dual-Functional Biomarker PLOD2 for Immunotherapy Stratification

Fei Xiao[1, #], Yi Guan[1, #], Huajie Song[1, *], Wan Xiang[2, *]

[1]Department of Urology, Renmin Hospital of Wuhan University, Wuhan, China

[2]Department of Biological Repositories, Zhongnan Hospital of Wuhan University, Wuhan, China

# These authors contributed equally to this work.

*Corresponding authors: Tel. +86-27-6781-2689, Fax: +86-27-6781-2892.

*Corresponding authors: Dr. Wan Xiang, email: xiangwan@whu.edu.cn, Dr. Huajie Song, email: huajie_S@163.com Tel. +86-27-6781-2689, Fax: +86-27-6781-2892.

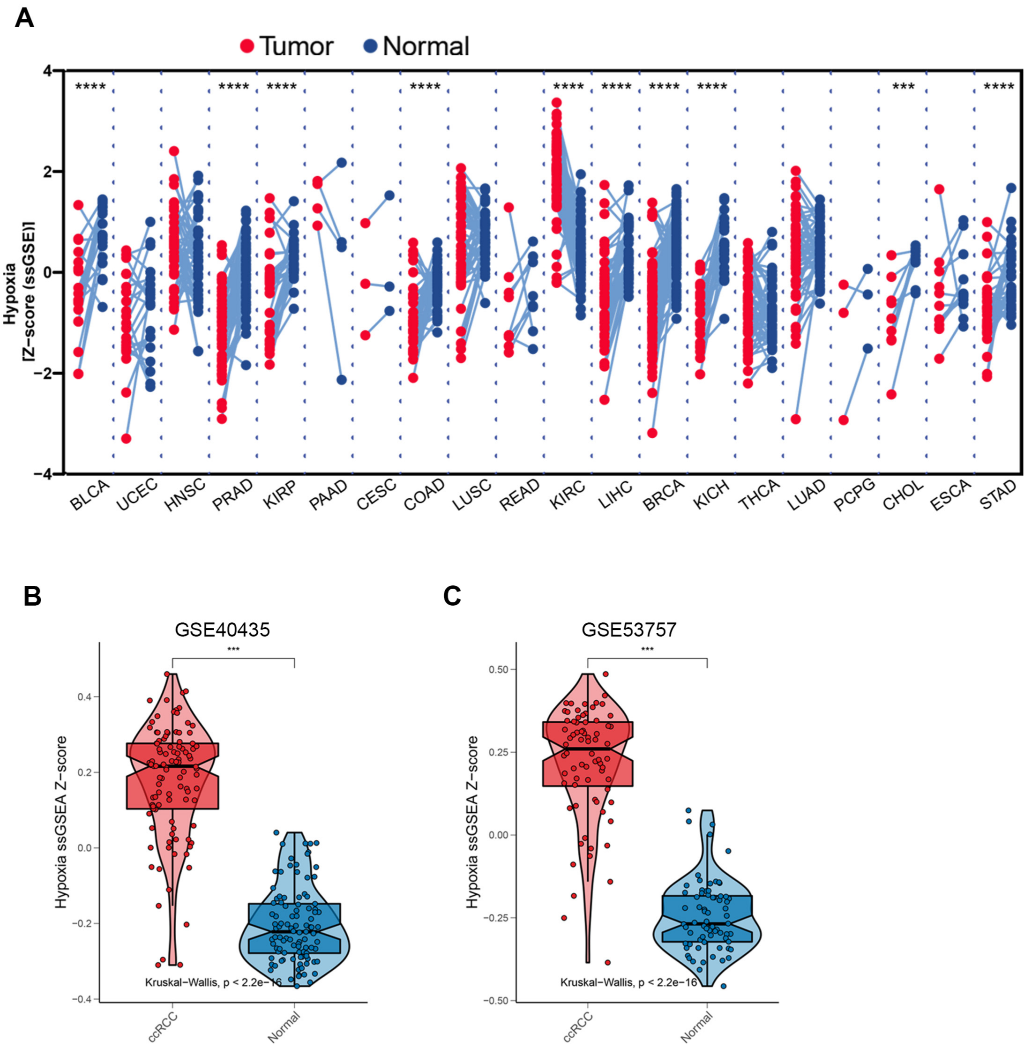

Fig S1. (A) All samples from the TCGA grouped by cancer with hypoxia enrichment scores. On the Y-axis is the hypoxia score calculated by ssGSEA based on TCGA data. Each point represents a sample. Student t-tests are used to compute p-values. (B-C) The hypoxia enrichment scores across all samples of independent datasets GSE40435 and GSE53757.

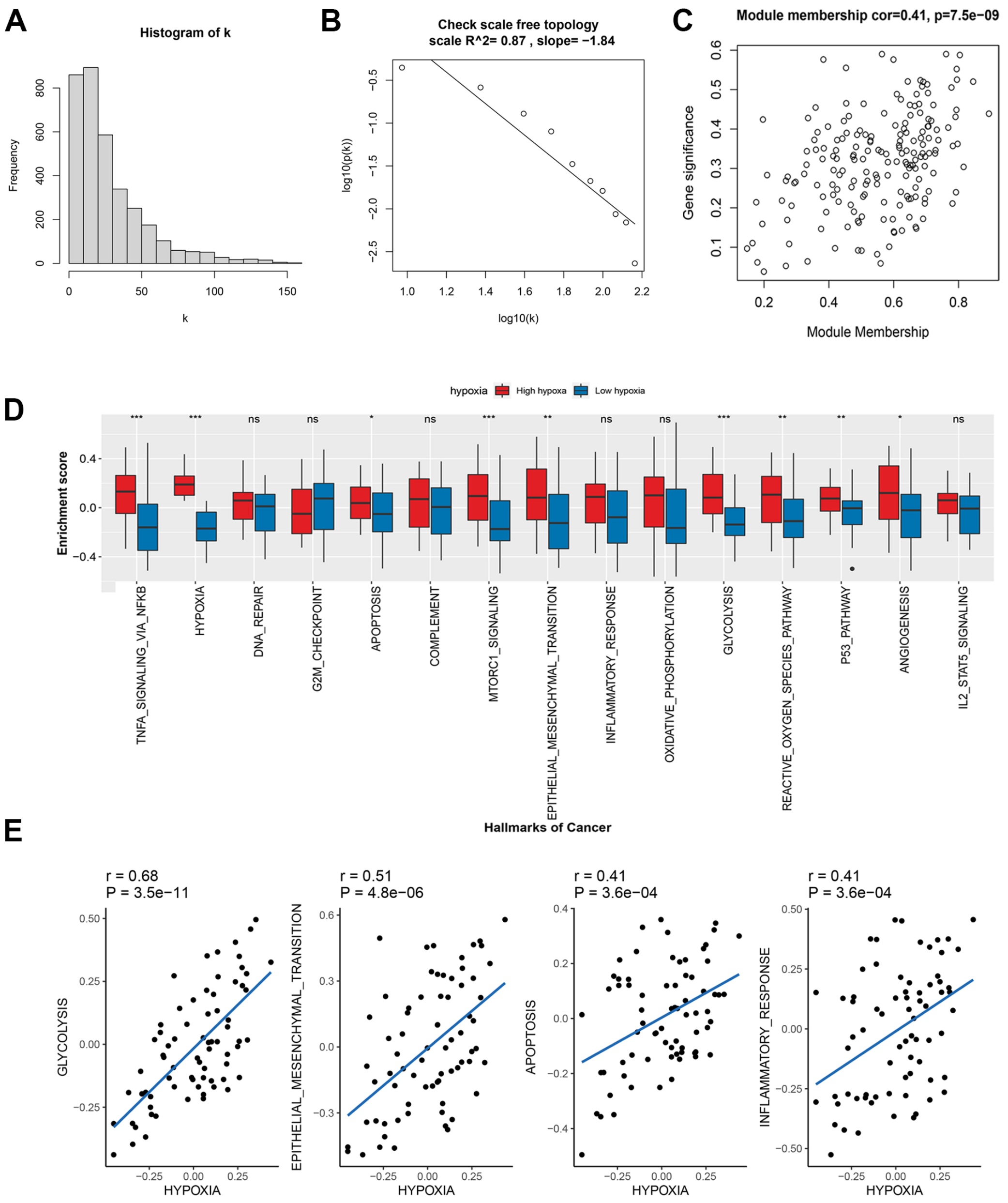

Fig S2. (A) Histogram of connectivity distribution (β = 4). (B) Checking the scale free topology (β = 4). (C) Scatter plot for correlation between gene module membership in the black module (hypoxia module) and gene significance. (D) Enrichment scores of cancer hallmarks between low and high hypoxia groups in dataset GSE53757. (E) Correlation between hypoxia level with glycolysis, epithelial mesenchyme transition, apoptosis, and inflammatory response in GSE53757.

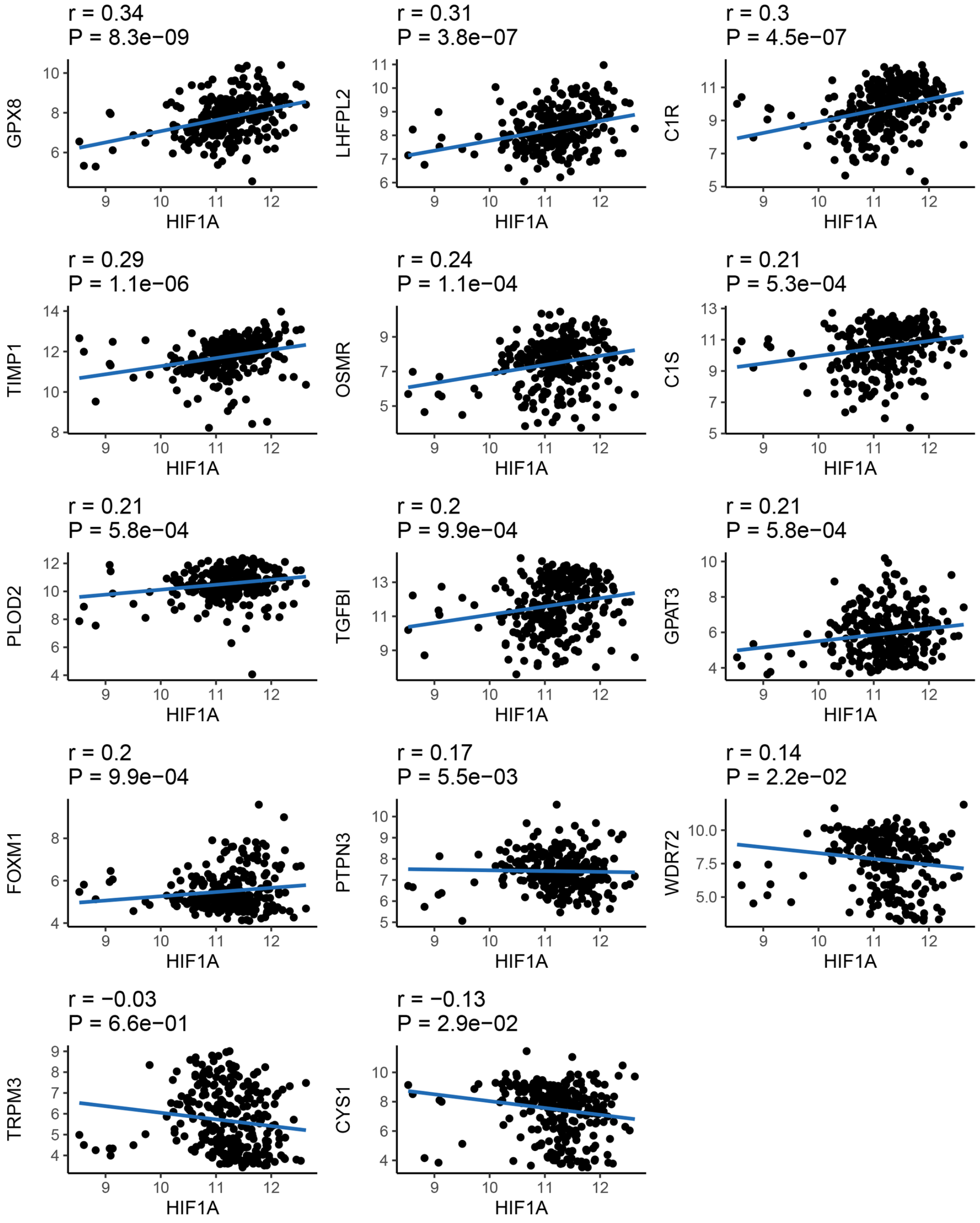

Fig S3. Correlation of HIF1A expression with the hypoxia hub genes expression in GSE73731.

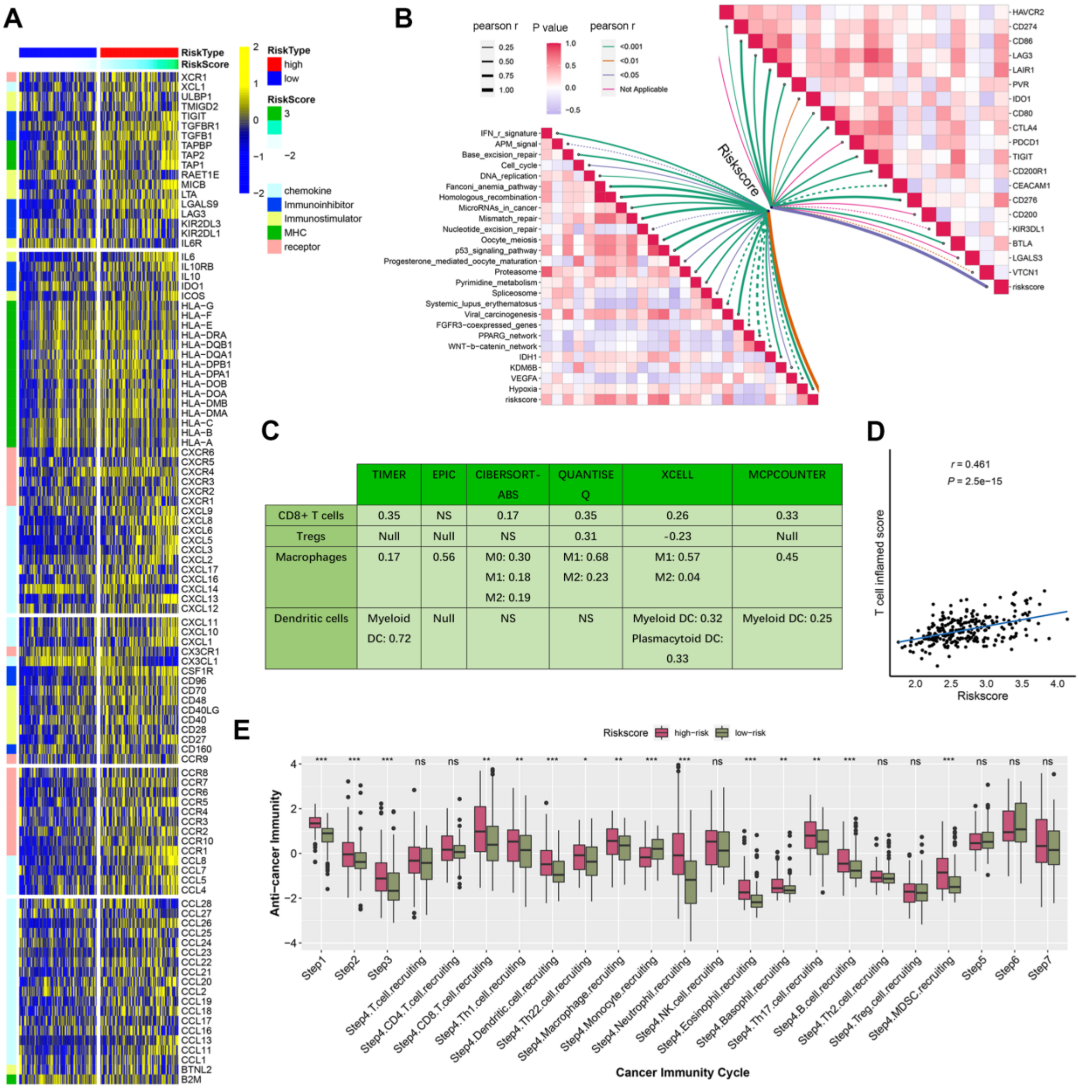

	TIMER	EPIC	CIBERSORT-ABS	QUANTISEQ	XCELL	MCPCOUNTER
CD8+ T cells	0.35	NS	0.17	0.35	0.26	0.33
Tregs	Null	Null	NS	0.31	-0.23	Null
Macrophages	0.17	0.56	M0: 0.30 M1: 0.18 M2: 0.19	M1: 0.68 M2: 0.23	M1: 0.57 M2: 0.04	0.45
Dendritic cells	Myeloid DC: 0.72	Null	NS	NS	Myeloid DC: 0.32 Plasmacytoid DC: 0.33	Myeloid DC: 0.25

Fig S4. (A) Expression differences between high- and low-risk groups of 122 immunomodulators (chemokines, receptors, MHC, and immunostimulators) in validation dataset GSE73731. (B)A correlation between HRS and the enrichment scores of immune checkpoints and pathways predicted for immunotherapy. (C) Infiltration levels of five types of TIIC (CD8+ T cells, macrophages, Th1 cells, and dendritic cells) were correlated with HRS (seven independent methods were used). (D) Correlation between HRS and the pan-cancer T cell inflammatory score. (E) Correlation between HRS and cancer immunity steps. (*P < 0.05; **P < 0.01; ***P < 0.001).

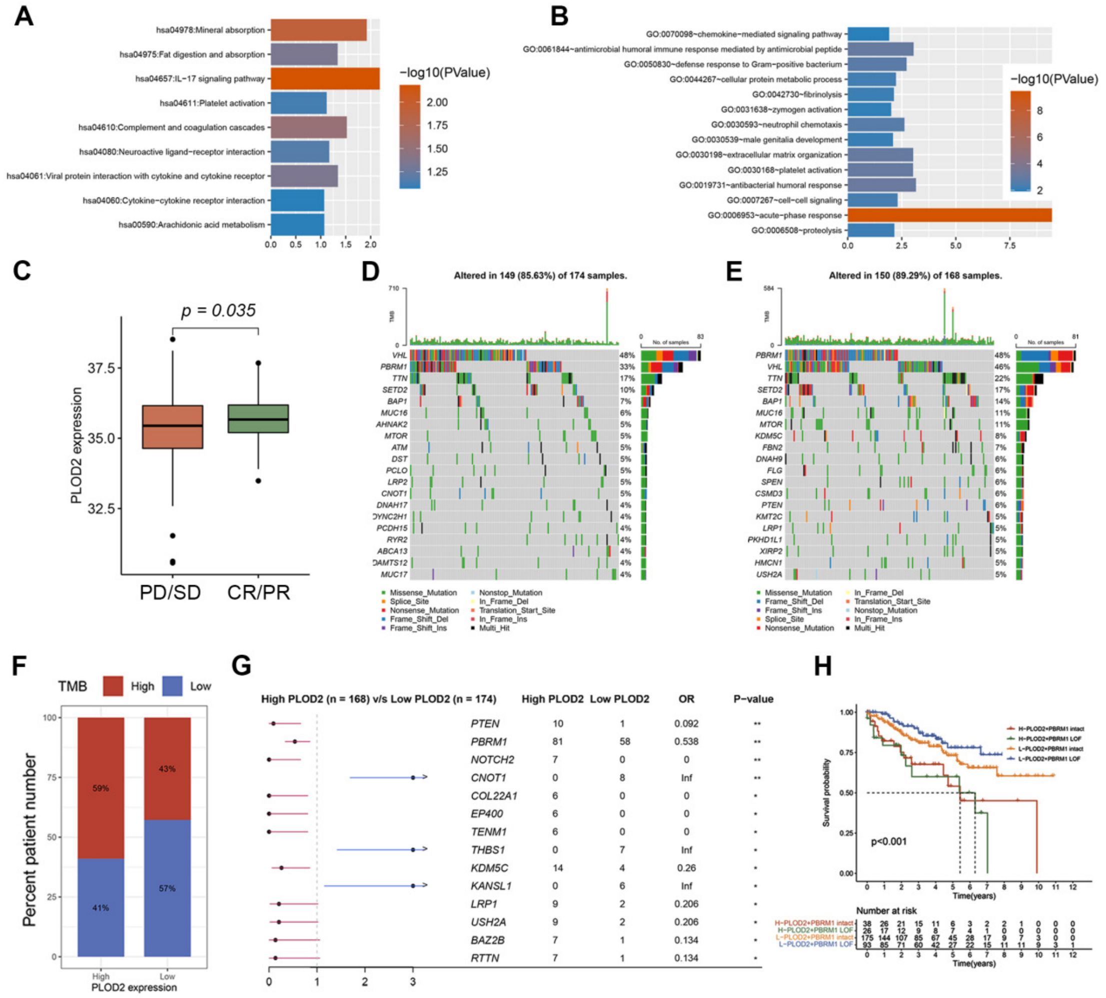

Fig S5. (A) Correlations between PLOD2 and the enrichment scores of immunotherapy-predicted pathways. (B) Correlation between PLOD2 and cancer immunity steps. (C) Expression differences between high- and low-PLOD2 groups of immunomodulators (chemokines, receptors, MHC, and immunostimulators) in TCGA KIRC. (D) Correlation between expression of PLOD2 and immune checkpoints. (E) Difference of PLOD2 expression between PBRM1 LOF (loss of function) and intact groups.

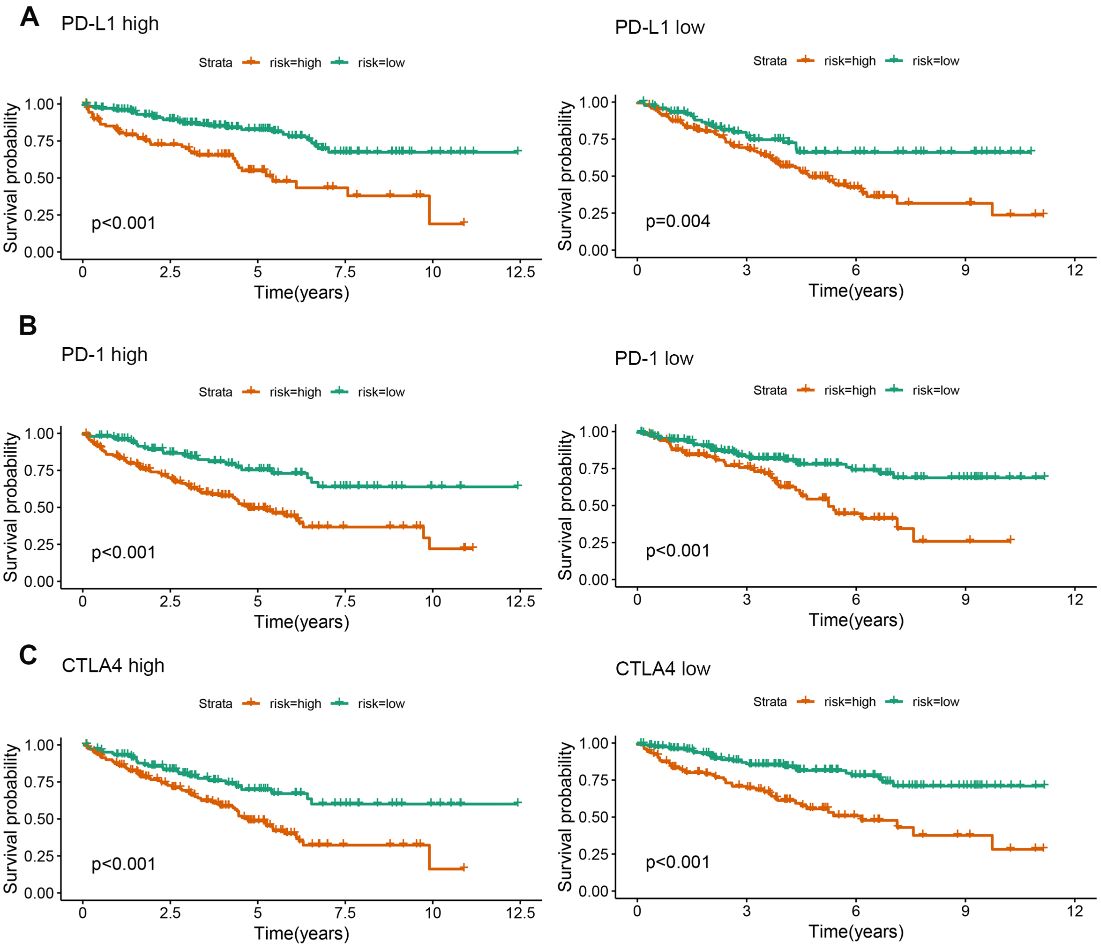

Figure S6. Kaplan-Meier curves demonstrating HRS stratification in PD-1/PD-L1/CTLA-4 expression subgroups.

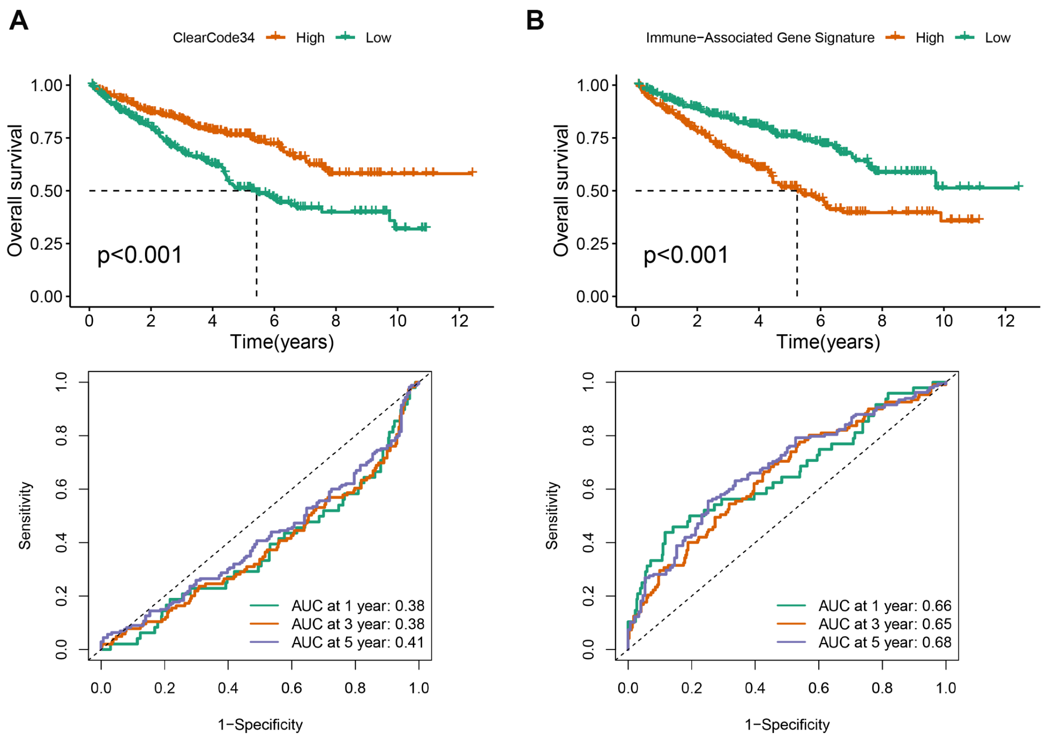

Figure S7. (A-B) Kaplan-Meier analysis and ROC curves demonstrated the predictive accuracy of ClearCode34 and immune-related signature stratification for viability in he entire TCGA collection.

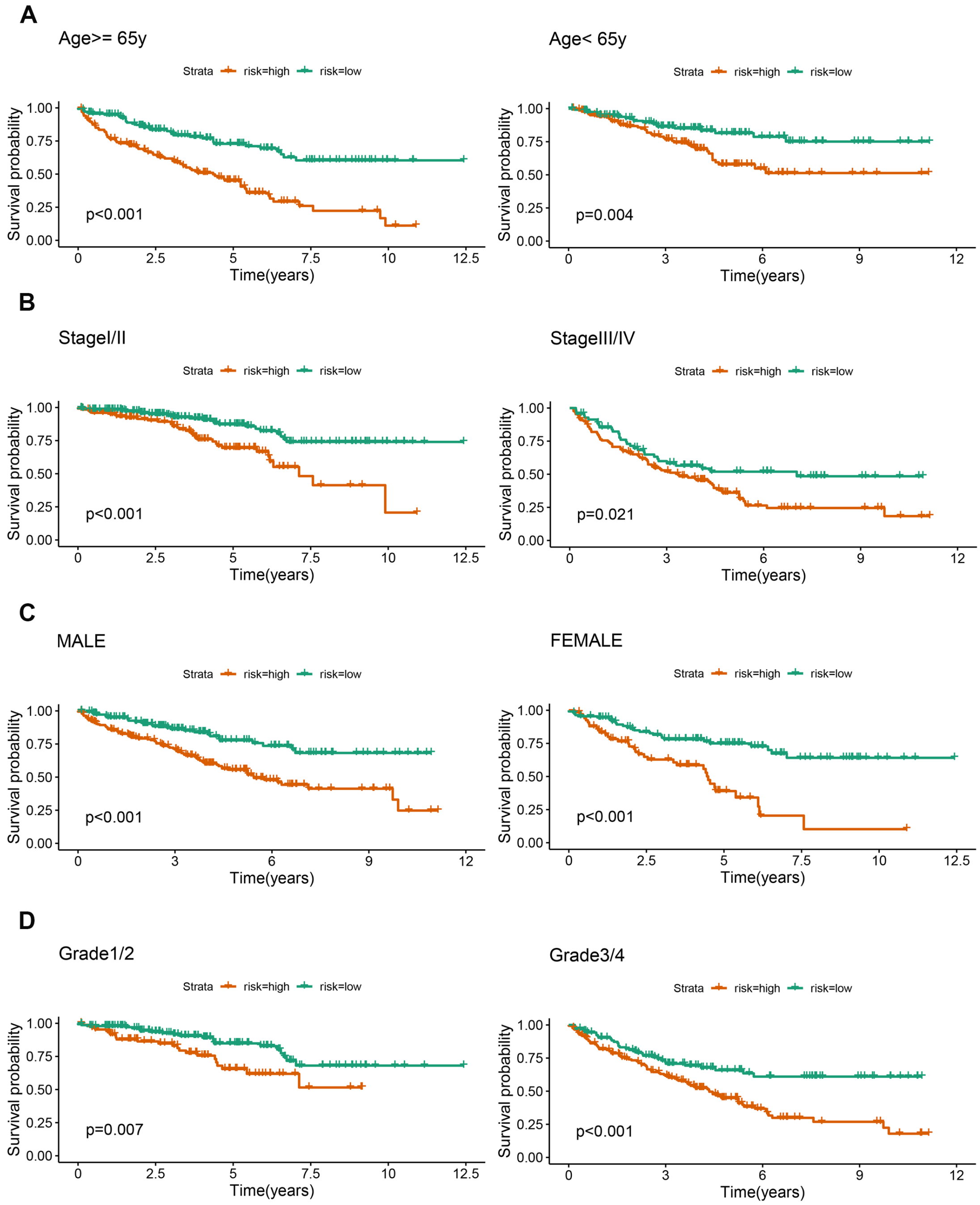

Figure S8. Kaplan-Meier curves demonstrating HRS stratification in different age, gender, tumor stage and grade subgroups.

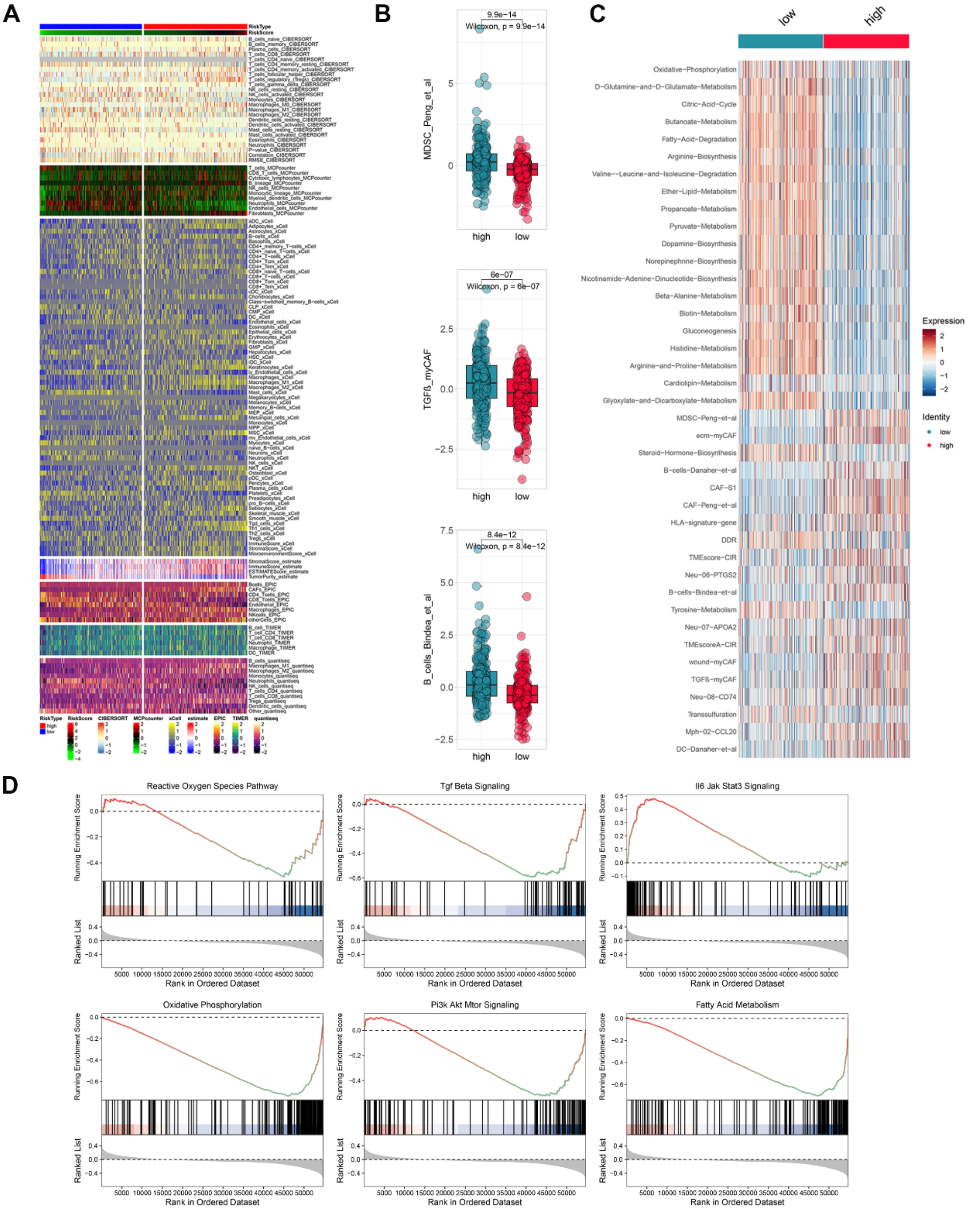

Figure S9. (A) Heatmap for immune responses based on CIBERSORT, ESTIMATE, MCPcounter, ssGSEA, EPIC and TIMER algorithms among high and low HRS groups. (B) Comparation of immune-related signatures such as MDSC, TGF-β and B cells between high and low HRS groups. (C) Heatmap for immune-related signatures among high and low HRS group. (D) GseaVis analysis confirmed enriched metabolic and immune evasion terms in high-HRS group.
